# Incidence of coronary drug-eluting stent fracture: A systematic review and meta-analysis

**DOI:** 10.3389/fcvm.2022.925912

**Published:** 2022-08-23

**Authors:** Yang Chen, Dandan Li, Yanhui Liao, Xiongda Yao, Yuehua Ruan, Kai Zou, Hanhui Liao, Jingwen Ding, Hao Qin, Zuozhong Yu, Yuanbin Zhao, Longlong Hu, Renqiang Yang

**Affiliations:** ^1^Department of Cardiovascular Medicine, The Second Affiliated Hospital of Nanchang University, Nanchang, China; ^2^Department of Neurology, The Second Affiliated Hospital of Nanchang University, Nanchang, China

**Keywords:** stent fracture, drug-eluting stent, meta-analysis, meta-regression, incidence

## Abstract

**Background:**

Reported evidence of coronary stent fracture (CSF) has increased in recent years. The purpose of this study was to determine reliable estimates of the overall incidence of CSF.

**Methods and results:**

The MEDLINE, Embase and Cochrane databases were searched until March 18, 2022. Pooled estimates were acquired using random effects models. Meta-regression and subgroup analysis were used to explore sources of heterogeneity, and publication bias was evaluated by visual assessment of funnel plots and Egger’s test. Overall, 46 articles were included in this study. Estimates of CSF incidence were 5.5% [95% confidence interval (CI): 3.7–7.7%] among 39,953 patients based on 36 studies, 4.8% (95% CI: 3.1–6.8%) among 39,945 lesions based on 29 studies and 4.9% (95% CI: 2.5–9.4%) among 19,252 stents based on 8 studies. There has been an obvious increase in the incidence of CSF over the past two decades, and it seems that the duration of stent placement after stent implantation has no impact on incidence estimation.

**Conclusion:**

The incidence of CSF was 5.5% among patients, 4.8% for lesions and 4.9% for stents and increased over the past 20 years. The duration of stent placement after stent implantation was found to have no impact on the incidence of CSF, but drug-eluting stent (DES) types and right coronary artery (RCA) lesions influenced the pooled incidence.

**Systematic review registration:**

[https://www.crd.york.ac.uk/prospero/display_record.php?ID=CRD42022311995], identifier [CRD42022311995].

## Introduction

Coronary drug-eluting stents (DES) are widely used in the interventional treatment of coronary heart disease. The material, structure, technology, and thickness of the steel beam and the drug loading model of the stent have changed significantly (1). With the maturity of interventional technology, coronary stents are used not only for simple A-type lesions but also widely used for C-type complex lesions, such as twisted angulation, calcification, and chronic total occlusion (2). Due to pulsation of the heart, metal fatigue, damage, or even coronary stent fracture (CSF) could occur in the coronary stent, which is affected by persistent complicated mechanical functions.

Especially in recent years, the thinner steel beam thickness in the new generation of alloy stents and the wide use of intraluminal imaging technology have led to a significant increase in the incidence of CSF in clinical practice (3–5). In addition, serious clinical cardiovascular events such as in-stent restenosis (ISR), stent thrombus (ST), coronary artery aneurysm and coronary perforation induced by CSF have threatened patients’ health (6–8). In addition, trials assessing the incidence of CSF and related risk factors for CSF have recently been published and added to the evidence base. There is a need for an update on the incidence of CSF and predictive factors of CSF.

Therefore, a systematic review and meta-analysis were performed to highlight the incidence of CSF and to summarize the current knowledge of related factors for CSF. We hypothesized that we could identify the incidence of CSF and the risk factors for CSF.

## Materials and methods

This systematic review and meta-analysis were performed according to Preferred Reporting Items for Systematic Reviews and Meta-Analyses (PRISMA) and Meta-Analysis of Observational Studies in Epidemiology (MOOSE) guidelines. The complete details for this meta-analysis, including the electronic search strategy, objectives, criteria for study selection, eligibility, data collection, and assessment of study quality, are available in the International Prospective Register of Systematic Reviews (PROSPERO) (CRD42022311995) on 25 March 2022.^1^

## Literature search and data extraction

We searched the Cochrane Central Register of Controlled Trials (CENTRAL; internet), MEDLINE, and Embase databases from the inception dates to 18 March 2022 using keywords and Medical Subject Headings (MeSH) terms relevant to the exposure of interest to collect data for the systematic review and meta-analysis: “Strut Fracture,” “Strut Fractures,” “Strut Fractured,” “Strut Fracturing,” “Strut Crack,” “Strut Cracked,” “Strut Cracks,” “Strut Cracking,” “Stent Fracture,” “Stent Fractured,” “Stent Fractures,” “Stent Fracturing,” “Stent Crack,” “Stent Cracked,” “Stent Cracks,” “Stent Cracking,” “DES Fracture,” “DES Fractures,” “DES Fractured,” “DES Fracturing,” “DES Crack,” “DES Cracked,” “DES Cracks,” “DES Cracking,” “BMS Fracture,” “BMS Fractures,” “BMS Fractured,” “BMS Fracturing,” “BMS Crack,” “BMS Cracked,” “BMS Cracks,” and “BMS Cracking.” There were no language restrictions. Clinical randomized trials, controlled before-and-after studies, case control studies, and prospective and retrospective cohort studies were eligible for inclusion in the review. Cross-sectional studies, repeat publications of the same analysis or dataset, case reports, conference abstracts, opinion pieces, books or gray literature were excluded. To acquire qualitative/quantitative analyses, inclusion and exclusion criteria were summarized according to the PICOS (Population, Intervention, Comparator, Outcomes, and Study) approach (Supplementary Table 1). Two researchers (YC and DDL) independently screened titles and abstracts. Disagreements were resolved by consensus. The authors looked through the possible relevant articles for inclusion in the final analysis after excluding non-relevant studies. Supplementary Records of selected studies were also evaluated under available conditions. Data were extracted from the included studies in a standardized form for the assessment of study quality and evidence synthesis. Extracted information included year of publication, study type, study period, population, sample size, participant demographics, baseline characteristics, type of DES and postprocedural outcomes.

## Outcomes and analyses

The primary outcome of this meta-analysis was the incidence of CSF. CSF was considered and recorded in the research analysis according to reports of the use of plain fluoroscopy, computed tomography (CT), coronary angiography (CAG), intravascular ultrasound (IVUS) or optical coherence tomography (OCT) in the selected studies. The review was performed in accordance with instructions given in the Cochrane Handbook for Systematic Reviews of Intervention.

## Risk of bias

Quality was assessed using the Cochrane Collaboration’s tool for assessing the risk of bias in randomized controlled trials (RCTs). For non-RCTs, the modified Newcastle–Ottawa quality assessment scale was used to assess the quality of the included cohort studies and case control studies. The total score for the modified Newcastle–Ottawa scale for cohort studies is a maximum of nine stars overall with a minimum of zero. A study was considered high quality if it achieved 7 out of 9 stars and moderate if it achieved 5 out of 9 stars (Supplementary Table 2). Overall quality was independently determined by each reviewer, and any discrepancies were resolved by consensus.

### Statistical analysis

Review Manager (RevMan, version 5.4) and the R package for Windows (version 4.1.0) (meta package) were used to perform all the meta-analyses. Random effects meta-analyses were performed using the proportions of patients who experienced CSF as the outcome of interest. Because of the anticipated high degree of heterogeneity, predominantly among non-RCTs, an inverse variance (DerSimonian–Laird) random effects model was applied. Following the identification of each study to be included, precise event rates were noted from the reported results in all cases. After transforming the event parameters to a normal distribution, an appropriate estimation method was chosen to estimate the original rate (9). Pooled effect estimates were expressed as risk ratios with 95% confidence intervals (CIs). Heterogeneity was explored within each meta-analysis using a chi-squared test with significance set at *P* < 0.10. We expressed the percentage of heterogeneity attributable to variation rather than to chance as *I*^2^ (10). Heterogeneity was defined as follows: *I*^2^ = 25–49%, low heterogeneity; *I*^2^ = 50–74%, moderate heterogeneity; and *I*^2^ > 75%, high heterogeneity (10). Based on the results of Cochran’s Q test and Higgins *I*^2^, the random effects method took into account the variability among the included studies to be as conservative as possible. A subgroup analysis was performed to investigate the relationship of DES types, follow-up evaluation modes or type of included studies with CSF incidence. Meta-regression models were performed by applying inverse variance weighting with a mixed effects model to explore the potential association between DES duration, study year, patient characteristics, lesion characteristics, stent characteristics and CSF incidence. Publication bias was assessed by visual assessment of funnel plots, Egger’s funnel plots and Egger’s test. Sensitivity analyses were conducted with R (metainf command) to calculate the combined proportion value and 95% confidence interval by excluding each included study separately.

## Results

### Study selection

**FIGURE 1:**
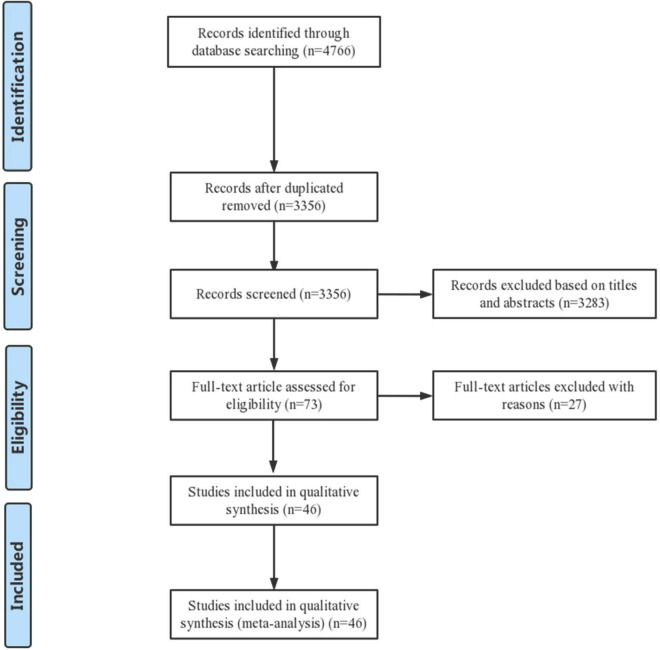
Flow diagram of included/excluded studies.

From the systematic review and meta-analysis search, 4,766 potentially eligible records were identified. Titles and abstracts of these records were screened for inclusion. The full texts of 73 records were read, and 46 studies were finally included in the data assessment (3–8, 11–50). Figure 1 shows the PRISMA flow diagram describing the study selection process along with the reasons for exclusion. In brief, a total of 21 prospective studies and 25 retrospective studies were ultimately included in the analysis.

### Study characteristics

Table 1 shows the main characteristics of the included studies. Table 2 summarizes the study participants’ characteristics. Lesion characteristics and follow-up results are presented in Table 3. All 46 selected papers were published after 2006. The number of included patients for each trial ranged from 30 to 6,555, with the number of included lesions ranging from 54 to 11,712. Among the 46 studies, 36 studies including 39,953 participants showed the incidence of CSF at the patient level, and 29 studies including 39,945 lesions showed the incidence of CSF at the lesion level. In addition, 8 studies including 19,252 stents showed the incidence of CSF at the stent level. Twenty studies included information on sirolimus-eluting stent (SES), and 4 included information on everolimus-eluting stent (EES); 15 studies evaluated CSF based on plain fluoroscopy or CAG, and 31 studies evaluated CSF based on further IVUS, OCT or CT. The total number of included stents was not equal to the sum of the numbers of all kinds of DESs because some studies provided only total numbers rather than DES types in detail. The point incidence of CSF varied from 0 to 38.2% at the patient level, 0 to 38.2% at the lesion level, and 1 to 22% at the stent level.

**TABLE 1:** Characteristics of included studies.

References	Study period	Study design	Inclusion criteria	Exclusion criteria	Definition of CSF	Pathway detecting CSF
Kim et al. (11)	2003–2005	Retrospective non-randomized clinical trial	SES implantation	Deficiency of follow-up CAG	Complete fracture	CAG
Okumura et al. (14)	2004–2005	Prospective non-randomized clinical trial	Unstable and stable angina; a target lesion in a native coronary artery; elective stent implantation; agreement to follow-up CAG	na	Significant disappearance of stent struts	CAG, IVUS
Aoki et al. (13)	2004–2005	Prospective non-randomized clinical trial	SES implantation	AMI, intolerance of aspirin or ticlopidine, planned surgery requiring antiplatelet therapy withdrawal within 3 months; inappropriate vessel size for SES	Complete separation	CAG, IVUS
Lee et al. (12)	2003–2005	Retrospective non-randomized clinical trial	SES or PES implantation, underwent clinically driven repeat angiography	na	Minor (single strut fracture), moderate (fracture > 1 strut), and severe (complete separation of stent segments)	CAG
Yamada et al. (16)	2004–2005	Prospective non-randomized clinical trial	SES implantation	Deficiency of follow-up angiography	Stent bending with separation of stent struts or disappearance of stent struts	CAG, IVUS
Chung et al. (15)	2003–2005	Retrospective non-randomized clinical trial	DES implantation	Deficiency of follow-up angiography	Interrupted connection of stent struts or fewer visible stent struts than normally looking stented area	CAG, IVUS
Kim et al. (26)	2004–2005	Prospective randomized clinical trial	Long coronary lesions (≥ 25 mm)	Death, insufficient clinical follow-up, or loss of repeat angiography	Minor (single strut fracture), moderate (fracture of >1 strut), and severe (complete separation of stent segments).	CAG
Maehara et al. (25)	2005–2007	Prospective randomized clinical trial	STEMI within 12 h of symptom onset	Poor image quality, no final IVUS image, or other reasons	Absence of struts over more than one third of the stent circumference	CAG, IVUS
Ino et al. (24)	2004–2007	Prospective non-randomized clinical trial	SES implantation in *de novo* and ISR lesions	STEMI who underwent pPCI; planned surgery requiring antiplatelet therapy withdrawal; intolerance of aspirin or ticlopidine; inappropriate vessel size for SES	Complete and partial stent fracture.	CAG
Hecht et al. (27)	na	Retrospective non-randomized clinical trial	Patients with stents implantation received CTA and their CAG data was available	na	Partial or complete separation of stent segments	CAG
Lee et al. (23)	2003–2008	Retrospective non-randomized clinical trial	DES implantation	Deficiency of follow-up angiography	Complete/incomplete separation of the stent strut. TypeI-IV: type I, a single strut fracture only; type II, multiple strut fractures at different sites; type III, complete transverse SF without displacement of fractured fragments more than 1 mm during the cardiac cycle; type IV, complete transverse linear type III fracture with stent displacement.	Fluoroscopy, IVUS
Fukuda et al. (22)	2003–2007	Retrospective non-randomized clinical trial	Patients with angina pectoris or positive stress test and *de novo* lesions in native coronary vessels who were implanted with a SES	AMI; PCI for ISR	na	CAG, IVUS
Umeda et al. (21)	2004–2005	Prospective non-randomized clinical trial	Successful SES implantation	Deficiency of follow-up angiography	Complete separation of stent or single or multiple stent strut fracture.	CAG, fluoroscopy, IVUS
Popma et al. (20)	2001	Prospective randomized clinical trial	Mild or moderately complex coronary artery narrowings with lesions 15–30 mm in length and a reference vessel 2.5–3.5 mm in diameter.	MI (<24 h); a totally occluded target lesion; a target lesion in an ostial or bifurcation location.	Popma classification*. TypeI-IV.	CAG
Kandzari et al. (19)	na	Prospective non-randomized clinical trial	SES implantation, TCO revascularization.	na	Discontinuity of stent architecture within the originally stented segment.	CAG
Yang et al. (18)	2005–2006	Retrospective non-randomized clinical trial	SES implantation, received follow-up coronary angiography	na	Complete separation of the stent segments and/or the absence of a stent strut	CAG
Kim et al. (17)	2003–2006	Retrospective non-randomized clinical trial	SES implantation	na	Complete or partial separation of stents.	CAG, IVUS
Ino et al. (29)	2004–2007	Prospective non-randomized clinical trial	SES implantation in *de novo* and ISR lesions	STEMI who underwent pPCI; planned surgery requiring antiplatelet therapy withdrawal; intolerance to aspirin or ticlopidine; inappropriate vessel size for SES.	Complete or partial separation of the stent. Type I-IV (Popma classification*).	CAG
Kawai et al. (28)	2002–2006	Prospective non-randomized clinical trial	Successful implantation with SES or BX-BMS	Deficiency of follow-up angiography between 6 and 9 months after PCI; total stent length greater than 40 mm	Complete separation of stent or single or multiple stent strut fracture.	CAG, fluoroscopy, IVUS
Park et al. (34)	2000–2009	Retrospective non-randomized clinical trial	Bx velocity stent implantation, SES implantation and received follow up CAG	Intolerant to aspirin, ticlopidine, or clopidogrel	Complete separation of the stent segments and/or the absence of a stent strut. Partial (only one of the inner or outer struts was separated) and complete (both the inner and outer struts were disconnected) types.	CAG, fluoroscopy
Park et al. (33)	2004–2007	Retrospective non-randomized clinical trial	Received PCI and underwent CAG follow-up	Deficiency of follow-up angiography between 6 and 12 months post PCI	Visible interrupted connection of stent struts or fewer visible stent struts than normal looking stented area.	CAG, IVUS
Serikawa et al. (32)	2004–2008	Prospective non-randomized clinical trial	AP or ACS treated with SES implantation	Deficiency of follow-up angiography 8 months after stenting	Complete discontinuation of the stent. Type III-IV (Popma classification*).	x ray
Umeda et al. (31)	2004–2006	Prospective non-randomized clinical trial	Underwent successful implantation with only SES	Deficiency of follow-up angiography between 6 and 9 months after stenting	Complete separation of stent or single or multiple stent strut fracture.	CAG, plain fluoroscopy, IVUS
Ino et al. (30)	2004–2007	Prospective non-randomized clinical trial	SES implantation in *de novo* and ISR lesions	STEMI who underwent pPCI; planned surgery requiring antiplatelet therapy withdrawal; intolerance of aspirin or ticlopidine/clopidogrel; inappropriate vessel size for SES.	Complete or partial separation of the stent.	Plain fluoroscopy
Davlouros et al. (38)	2002–2008	Retrospective non-randomized clinical trial	Stent implantation, residence at a distance less than 100 km away from the institute, telephonic contact	Lost at follow up, death, reported coronary re-intervention following the initial procedure, without consent	Discontinuity of stent struts on fluoroscopy. Type I-IV (Popma classification*).	FPDD cinefluoroscopy, IVUS, OCT
Kim et al. (37)	2003–2007	Retrospective non-randomized clinical trial	PCI with implantation of SES	Deficiency of follow-up angiography	Complete or partial separation of the stent. Type I-IV (Popma classification*).	CAG, IVUS
Park et al. (36)	2003–2009	Retrospective non-randomized clinical trial	Newer DES platforms/Cipher Bx lesions implantation	Deficiency of follow-up angiography	Complete fracture (complete separation of stent segments) or partial fracture (single or multiple stent SF without separation of segment)	Fluoroscopy, CAG
Kuramitsu et al. (35)	2010–2011	Prospective non-randomized clinical trial	Underwent successful implantation with only EES	Deficiency of follow-up angiography between 6 and 9 months after stenting	Complete or partial separation of stent.	Fluoroscopy without contrast injection or IVUS
Hakim et al. (41)	2005–2010	Retrospective non-randomized clinical trial	IVUS-guided stent implantation with IVUS follow-up data available >1.5 years after implantation without an intervening intervention	na	Partial stent fracture (absence of stent struts for 1/3 of the stent circumference) or complete fracture (absence of stent struts for the entire circumference of the stent).	IVUS
Hara et al. (40)	2004–2005	Retrospective non-randomized clinical trial	SES implantation	AMI, intolerance to antiplatelet therapy, planned surgery within 3 months requiring withdrawal of antiplatelet therapy, inappropriate vessel size for stent implantation, without 8 months follow-up and 5-year clinical follow up	Complete separation of a previously contiguous stent	CAG, IVUS
Kozuma et al. (39)	2010–2010	Prospective randomized clinical trial	PCI using DES	Withdrew consent	Partial strut fracture or complete separation of the stented segment.	CAG
Inaba et al. (44)	2010–2012	Retrospective non-randomized clinical trial	Consecutive EES-treated lesions in patients who underwent IVUS follow-up who had either symptoms or evidence of ischemia by non-invasive imaging	na	Complete stent fracture– defined as separation of the stent into ≥ 2 pieces by image slices with no visible stent struts. Partial stent fracture– defined as the absence of struts over ≥ 1/3 of the stent circumference with separation of the proximal and distal fragments.	IVUS
Kuramitsu et al. (43)	2011–2012	Prospective non-randomized clinical trial	Treated with only BES	Deficiency of follow-up angiography between 6 and 9 months after PCI	Complete or partial separation of stent.	Fluoroscopy without contrast injection, IVUS, OCT
Ito et al. (42)	2004–2009	Retrospective non-randomized clinical trial	SES implantation, undergo MSCT between 6 and 18 months	TLR performed within 3 months after follow up MSCT, insufficient stent image quality, lost follow up	Complete gap upon visual inspection	MSCT
Pracon et al. (45)	2002	Prospective randomized clinical trial	Patients with a single *de novo* native coronary artery lesion	Contrary to the protocol, IVUS imaging was not performed, the pullback was not consistent throughout the length of the stent, the imaging quality was not adequate.	Complete separation	CAG, IVUS
Ohya et al. (46)	2002–2005	Retrospective non-randomized clinical trial	SES implantation, follow-up angiography within 1 year after the initial procedure	Patients having death or ST within 1 year were excluded; lesions with TLR and lesions lost to follow-up within 1 year were excluded	Complete separation of stent segments or stent struts	CAG
Kuramitsu et al. (3)	2012–2013	Prospective non-randomized clinical trial	Treated only with PtCr-EES	Deficiency of follow-up angiography between 6 and 9 months after PCI	Complete or partial separation of stent.	Fluoroscopy, IVUS, OCT
Kan et al. (7)	2003–2014	Prospective non-randomized clinical trial	DES implantation; repeat angiography.	BMS use and poor quality of angiographic images	Complete or partial separation of the stent. Type I-IV (Popma classification*).	CAG, IVUS, OCT
Chung et al. (47)	2011–2013	Retrospective non-randomized clinical trial	Patients who underwent PCI with DES as well as follow-up CCTA due to recurrent angina	Small stent less than 3 mm in diameter, poor image quality such as severe motion artifact or beam hardening artifact were excluded	Complete or partial disruption of a stent strut	CCTA
Ohya et al. (4)	2003–2012	Retrospective non-randomized clinical trial	DES implantation	Deficiency of follow-up angiography within 1 year	Separation of stent segments or stent struts.	CAG
Miura et al. (6)	2010	Retrospective non-randomized clinical trial	EES implantation	Repeat EES implantation and the combined use of EES and other types of stent.	Complete separation of stent segments or stent struts	CAG
Kuramitsu et al. (5)	2004–2008	Retrospective non-randomized clinical trial	SES implantation	Other stent implantation and deficiency of follow up angiography	Complete or partial separation of stent segments. TypeI-IV(Popma classification*).	Fluoroscopy
Watanabe et al. (49)	2005–2013	Retrospective non-randomized clinical trial	Patients with *de novo* proximal RCA lesions who received DES implants	History of AMI, coronary artery bypass graft surgery, ISR, BMS implantation, plain balloon angioplasty, patients who had no angiographic follow-up data within 18 months after DES implantation.	Complete and partial SF	Fluoroscopy
Ge et al. (48)	2004–2014	Prospective non-randomized clinical trial	2nd DES implantation and subsequent angiographic follow-up	(1) BMS use, first-generation DES, mixed implantation of first-generation and second- generation DES, and (2) repeat angiography ≤ 30 days after index PCI, and poor quality of angiographic images.	Complete or partial separation of stent segments. TypeI-IV(Popma classification*).	CAG, IVUS, OCT
Blessing et al. (50)	2018–2019	Retrospective non-randomized clinical trial	CTO treated with bioengineered Combo Dual-Therapy CD34 Antibody-Covered SES (Combo DTS) and follow up	na	Strut fracture with stent deformation and intraluminal protruding stent strut	OCT
Schochlow et al. (8)	na	Retrospective non-randomized clinical trial	Newer-generation DES and follow-up OCT	na	Pattern 1: one single stacked strut; Pattern 2: two or more stacked struts without deformation; Pattern 3: deformation with evidence of isolated (malapposed) struts or groups of struts not fitting the normal circular geometry of the scaffold in one or more cross sections; Pattern 4: transection with malalignment of the stent segments with or without gap (at least 2 consecutive frames without any strut)	OCT

ACS, acute coronary syndrome; AMI, acute myocardial infarction; AP, angina pectoris; BES, biolimus-eluting stent; BMS, bare-metal stent; CAG, coronary angiography; CCTA, coronary computed tomography angiography; CSF, coronary stent fracture; CTO, chronic total occlusion; DES, drug-eluting stent; EES, everolimus-eluting stent; FPDD, flat panel digital detector; ISR, in-stent restenosis; IVUS, intravascular ultrasound; MI, myocardial infarction; MSCT, multislice computed tomography; OCT, optical coherence tomography; PCI, percutaneous transluminal coronary intervention; PES, paclitaxel-eluting stent; pPCI, primary percutaneous transluminal coronary intervention; SES, sirolimus-eluting stent; ST, stent thrombosis; STEMI, ST-segment elevation myocardial infarction; TCO, total coronary occlusion.

*Popma classification. TypeI-IV. Fractures were classified as isolated strut fractures (type I, single-strut fracture; type II, incomplete transverse fracture) and stent fracture (type III, complete transverse fracture without displacement; type IV, transverse fracture with displacement).

**TABLE 2:** Characteristics of participants in the included studies.

Study (author, year)	Number of patients	Number of lesions	Number of stents	Age (mean ± *SD*)	Type of stent	Male, *n*	HT, *n*	DM, *n*	Dyslipidemia, *n*
Kim et al. (11)	457	na	na	na	SES	na	na	na	na
Okumura et al. (14)	138	169	na	62.72 ± 0.74	SES	112	108	42	81
Aoki et al. (13)	256	307	na	64.9 ± 8.7	SES	221	183	75	178
Lee et al. (12)	530	na	na	na	SES, PES	na	na	na	na
Yamada et al. (16)	56	83	102	70.8 ± 10.7	SES	36	32	25	20
Chung et al. (15)	4,190 (3095SESs, 1095PESs)	na	na	na	SES, PES	na	na	na	na
Kim et al. (26)	415	415 (210SESs, 205PESs)	na	60.2 ± 9	SES, PES	232	139	142	118
Maehara et al. (25)	na	219	na	60.4 ± 13.1	PES	na	na	na	na
Ino et al. (24)	273	364	na	67 ± 10	SES	214	218	125	184
Hecht et al. (27)	143	na	384	na	DES	na	na	na	na
Lee et al. (23)	1,009	na	na	na	DES	na	na	na	na
Fukuda et al. (22)	227	227	na	65.7 ± 9.3	SES	151	164	126	158
Umeda et al. (21)	382	430	na	na	SES	292	230	126	217
Popma et al. (20)	305	na	na	na	SES	na	na	na	na
Kandzari et al. (19)	200	na	na	60.3 ± 11.0	SES	160	139	49	172
Yang et al. (18)	479	na	686	61.4 ± 9.8	SES	306	na	na	na
Kim et al. (17)	557	628	678	59.4 ± 9.8	SES	394	255	142	45
Ino et al. (29)	387	517	na	66.9 ± 10	SES	na	na	na	na
Kawai et al. (28)	416	478	na	68 ± 6.1	SES	324	270	133	245
Park et al. (34)	268	314	na	60.7 ± 10.9	SES	na	na	na	na
Park et al. (33)	3,315	na	na	63.8 ± 11.5	DES	na	na	na	na
Serikawa et al. (32)	1,079	1,228	na	na	SES	na	na	na	na
Umeda et al. (31)	793	874	na	65.6 ± 9.8	SES	634	532	334	495
Ino et al. (30)	399	537	na	68 ± 9.8	SES	317	317	183	266
Davlouros et al. (38)	145	na	200	62.5 ± 9	SES	117	96	86	87
Kim et al. (37)	1,054	na	na	na	SES	na	na	na	na
Park et al. (36)	1,742	2,140	na	65.8 ± 9.5	DES	1,196	1,126	580	na
Kuramitsu et al. (35)	1,035	1,339	na	69.7 ± 9.6	EES	782	852	476	786
Hakim et al. (41)	47	54	na	66.6 ± 10.9	SES, PES, and others	35	34	20	33
Hara et al. (40)	222	264	na	64.9 ± 8.8	SES	183	161	69	158
Kozuma et al. (39)	482 (235EESs, 247SESs)	557	na	69.5 ± 9.5	SES/EES	356	379	210	362
Inaba et al. (44)	136	177	na	na	EES	na	na	na	na
Kuramitsu et al. (43)	1,026	1,407	na	70.1 ± 9.8	BES (Nobori)	780	803	440	746
Ito et al. (42)	528	644	na	67.9 ± 9.4	SES	393	336	168	265
Pracon et al. (45)	125	246	na	62.74 ± 10.03	DES (Taxus)	87	79	40	87
Ohya et al. (46)	972	1,795	na	68.7 ± 10.8	SES	706	665	409	481
Kuramitsu et al. (3)	700	898	na	69.7 ± 9.7	PtCr-EES	511	562	320	580
Kan et al. (7)	6,555	10,751	16,482	64.16 ± 10.28	DES	4,882	4,862	2,219	4,364
Chung et al. (47)	374	na	535	60.3 ± 10.4	DES	295	207	107	125
Ohya et al. (4)	5,456	11,712	na	na	DES	na	na	na	na
Miura et al. (6)	636	1,081	na	69.6 ± 10.9	EES	466	483	253	395
Kuramitsu et al. (5)	868	1,144	na	67.6 ± 9.0	SES	551	543	349	385
Watanabe et al. (49)	131	131	na	70.5 ± 9.7	DES	89	105	48	95
Ge et al. (48)	3,411	5,560	4,639	64.1 ± 10.2	2nd DES	2,551	2,519	na	2,236
Blessing et al. (50)	30	na	na	67.21 ± 11.57	SES	na	na	na	na
Schochlow et al. (8)	160	na	185	65.5 ± 17.9	DES	119	123	54	84

BES, biolimus-eluting stent; DES, drug-eluting stent; EES, everolimus-eluting stent; PES, paclitaxel-eluting stent; SD, standard deviation; SES, sirolimus-eluting stent.

**TABLE 3:** Results of follow up in included studies.

References	Duration after stent implantation	RCA target lesion, *n*	Type B2 or C, *n*	Stent length, mm	Stent/lesion ratio	SF		
						Patient	Lesion	Stent
Kim et al. (11)	6–9 months (mean 7.2 months)	na	na	na	na	11	na	na
Okumura et al. (14)	8 months	35	69	24.4 ± 9.3	1.25 ± 0.25	na	4	na
Aoki et al. (13)	240 ± 43 days	88	247	31.2 ± 16.6	1.45 ± 0.72	8	8	na
Lee et al. (12)	na	na	na	na	na	10	na	na
Yamada et al. (16)	6 months	14	64	27.2 ± 11.9	1.23	3	3	3
Chung et al. (15)	9 months	na	na	na	na	26 (26SESs, 0PES)	na	na
Kim et al. (26)	6 months	108	na	41.0 ± 13.2	na	7	7 (6SESs, 1PESs)	na
Maehara et al. (25)	13 months	na	na	na	na	na	6	na
Ino et al. (24)	6–9 months (7.6 ± 1.8 months)	103	293	25.0 ± 10.7	na	18	18	na
Hecht et al. (27)	na	na	na	na	na	na	na	4
Lee et al. (23)	6–9 months (15.6 ± 11.6 months)	na	na	na	na	15	17	na
Fukuda et al. (22)	6–9 months	na	na	25.1 ± 10.5	1.36	0	0	na
Umeda et al. (21)	6.9 ± 2.5 months	132	335	24.5 ± 10.3	na	32	33	na
Popma et al. (20)	8 months	na	na	na	na	4	na	na
Kandzari et al. (19)	6 months	98	na	45.9 ± 23.63	na	32	na	na
Yang et al. (18)	6–9 months (7.8 ± 3.4 months)	238	na	35.6 ± 16.1	na	18	na	22
Kim et al. (17)	9 months (range 2–30 months)	133	573	27.9 ± 11.6	na	21	na	21
Ino et al. (29)	6–9 months	na	na	na	na	na	29	na
Kawai et al. (28)	6–9 months (7.1 ± 3.5 months)	129	279	21.4 ± 6.0	na	na	21	na
Park et al. (34)	14.0 ± 11.8 months	na	na	na	na	na	29	na
Park et al. (33)	6–12 months	na	na	na	na	24	28	na
Serikawa et al. (32)	8 months (7.6 ± 2.6 months)	na	na	na	na	100	117	na
Umeda et al. (31)	6–9 months (7.6 ± 4.0 months)	255	na	25.3 ± 11.2	1.30 ± 0.57	69	70	na
Ino et al. (30)	6–9 months	155	436	26.5 ± 13.0	1.2 ± 0.5	na	9	na
Davlouros et al. (38)	45.5 ± 15.7 months	60	100	18.1 ± 4.9	na	6	na	6
Kim et al. (37)	8–10 months (378 ± 89 days)	na	na	na	na	99	109	na
Park et al. (36)	11.6 ± 5.0 months	521	1,238	36.1 ± 13.3	1.16 ± 0.55	54	55	na
Kuramitsu et al. (35)	6–9 months[233 days (IQR 185–246 days)]	421	914	29.9 ± 18.5	na	39	39	na
Hakim et al. (41)	2.8 years (min 1.6 and max 4.5).	11	na	18.6 ± 7.1	1.2 ± 0.5	na	5	na
Hara et al. (40)	8 months	72	221	21.9 ± 12.3	1 ± 0.74	na	6	na
Kozuma et al. (39)	278 ± 63 days	167	462	24.0 ± 12.4	1.22 ± 0.50	4 (SESs)	na	Na
Inaba et al. (44)	441 ± 317 days	na	na	na	na	na	15	na
Kuramitsu et al. (43)	6–9 months [192 days (IQR 183–223 days)]	451	931	30.6 ± 18.5	na	57	58	na
Ito et al. (42)	6–18 months	208	584	28.6 ± 16	1.36 ± 0.71	39	44	na
Pracon et al. (45)	9 months	na	189 (B/C)	25.22 ± 9.43		0	0	na
Ohya et al. (46)	8 months	632	na	28.8 ± 0.51	1.28 ± 0.57	99	105	na
Kuramitsu et al. (3)	190 days (IQR 183–234 days)	326	533	24 ± 13.33	na	16	16	na
Kan et al. (7)	340 ± 2,488 days	3,669	na	38.1 ± 21.9	1.53 ± 0.70	803	1,852	3,630
Chung et al. (47)	na	132	na	33.5 ± 15.8	1.7 ± 1.0	43	na	50
Ohya et al. (4)	8 months	na	na	na	na	446	494	na
Miura et al. (6)	8 months	403	787	27.7 ± 15.9	1.29 ± 0.58	29	29	na
Kuramitsu et al. (5)	188 (IQR 175–239)	276	702	23.0 ± 11.11	na	64	66	na
Watanabe et al. (49)	1 year ± 6 months	131	na	na	na	50	50	na
Ge et al. (48)	255.7 ± 61 days	1,110	1,988	35.5 ± 20.6	1.63 ± 0.78	426	na	na
Blessing et al. (50)	189 days (range 157–615 days)	na	na	na	na	11	na	na
Schochlow et al. (8)	na	60	103	na	na	19	na	21

IQR, inter quartile range; PES, paclitaxel-eluting stent; RCA, right coronary artery; SES, sirolimus-eluting stent; SF, stent fracture.

### Risk of bias and study quality

The risk of bias of the included studies is summarized in the Supplemental Material. Quality assessment using the modified Newcastle–Ottawa scale for observational studies showed a low risk of bias with no low-quality study (Supplementary Table 2). There was no high risk of bias for RCTs in terms of random sequence generation, allocation concealment, blinding of participants or blinding of outcome assessment. The particulars of individual bias domains are shown in the risk of bias (Supplementary Figures 1, 2).

### Publication bias

Funnel plots for all the included studies showed symmetrical distributions, indicating no publication bias for the incidence of CSF at the patient and lesion levels (Supplementary Figures 9–12). However, a funnel plot for CSF incidence at the stent level showing an asymmetric distribution indicated likely publication bias (Supplementary Figures 13, 14). Furthermore, analysis using Egger’s test provided similar results at the patient, lesion and stent levels (*P* = 0.5619, *P* = 0.0690, *P* = 0.0008) (Supplementary Tables 6–8).

### Pooled incidence rates of coronary stent fracture

**FIGURE 2:**
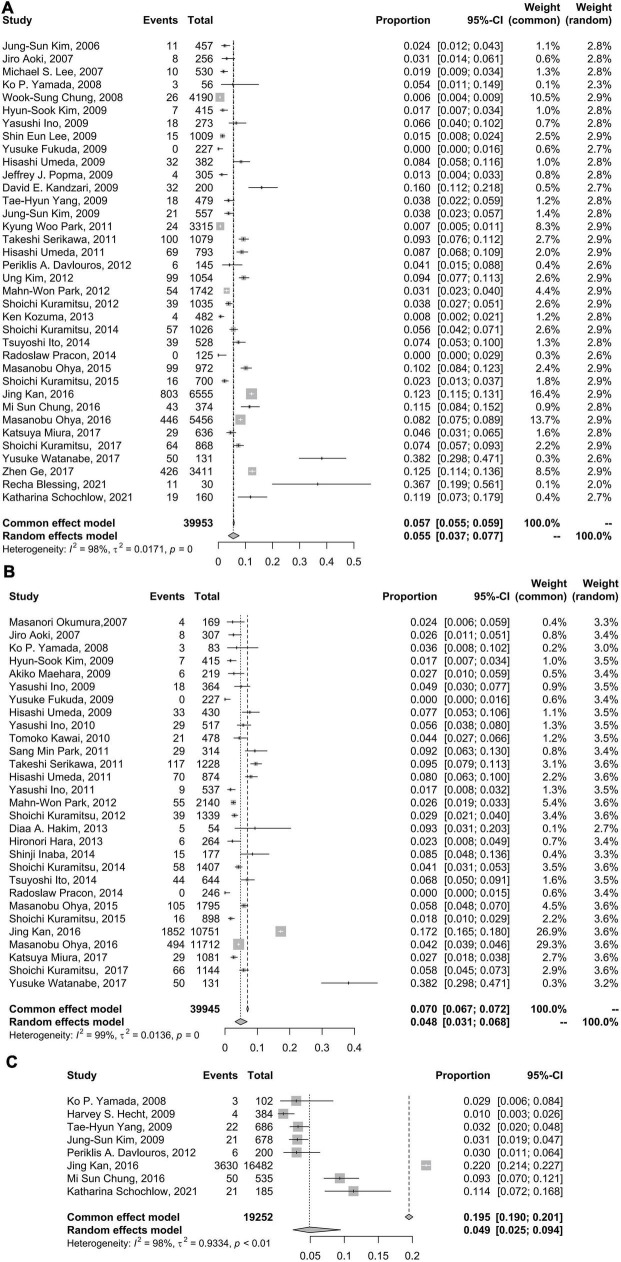
Forest plots of incidence of coronary stent fracture in patient level **(A)**, incidence of stent fracture in lesion level **(B)** and incidence of stent fracture in stent level **(C)**. CI, confidence interval.

The pooled incidence rate for CSF from 36 studies with 39,953 subjects (2,702 patients with CSF) was 5.5% (95% CI: 3.7–7.7%) using a random effects model because of a high level of heterogeneity (98%) (Figure 2). In comparison, the CSF incidence was 4.8% (95% CI: 3.1–6.8%) for 39,945 lesions (3,188 lesions with CSF) in 29 studies and 4.9% (95% CI: 2.5% to 9.4%) for 19,252 stents (3,757 stents with CSF) in 8 studies.

### Drug-eluting stent type

**FIGURE 3:**
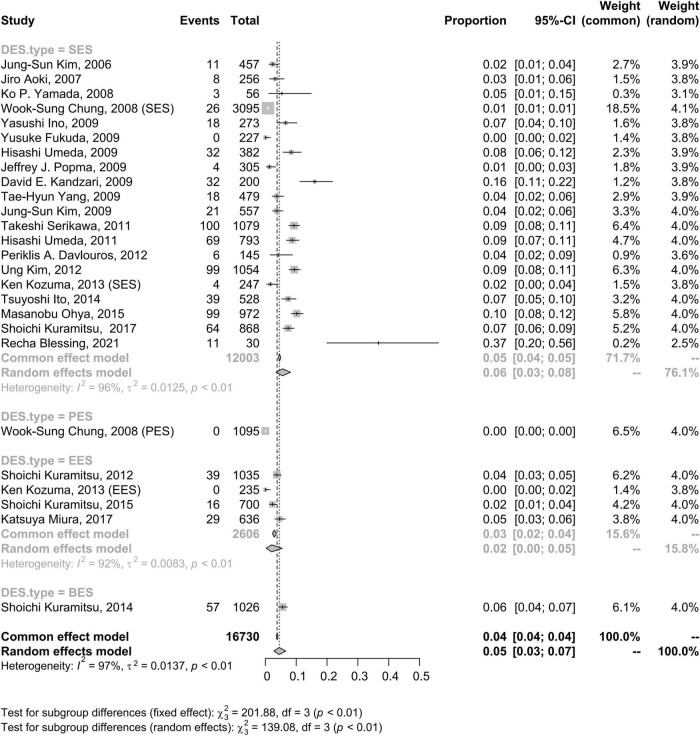
Forest plots of incidence of coronary stent fracture in patient level in different DES type. BES, biolimus-eluting stent; CI, confidence interval; DES, drug-eluting stent; EES, everolimus-eluting stent; PES, paclitaxel-eluting stent; SES, sirolimus-eluting stent.

The CSF incidence rates for sirolimus-eluting stents (SESs), paclitaxel-eluting stents (PESs), everolimus-eluting stents (EESs), and biolimus-eluting stents (BESs) were 6% (95% CI: 3–8%), 0% (95% CI: 0–0%), 2% (95% CI: 0–5%), and 6% (95% CI: 4–7%), respectively. Tests for subgroup differences in DES type with random effects showed remarkable differences (*p* < 0.01) (Figure 3).

### Follow-up evaluation modes

**FIGURE 4:**
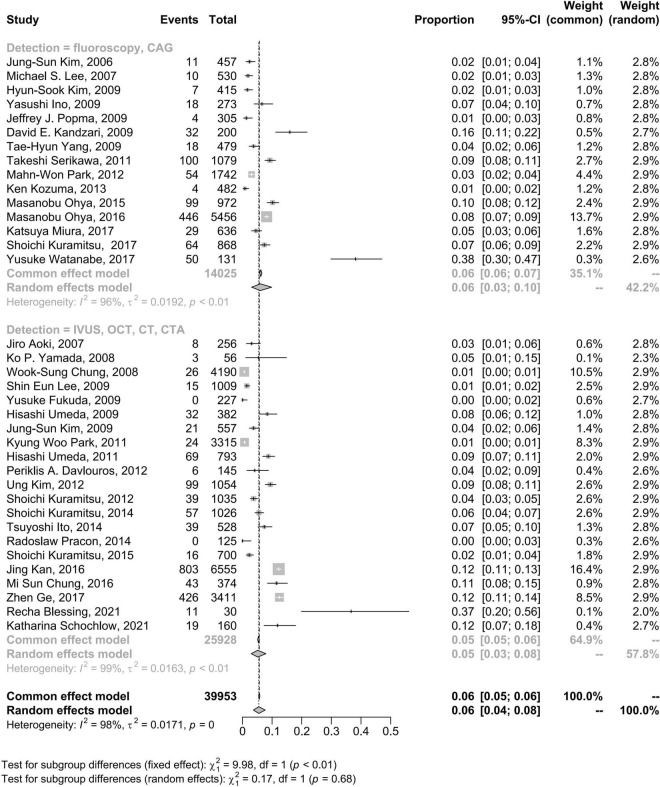
Forest plots of morbidity of coronary stent fracture in patient level in different follow up modes. CAG, coronary angiography; CI, confidence interval; CT, computed tomography; CTA, computed tomography angiography; IVUS, intravascular ultrasound; OCT, optical coherence tomography.

The incidence rate of CSF at the patient level was not significantly different when the follow-up evaluation was executed with IVUS, OCT or CT (5%, 95% CI: 3–8%) compared to when the follow-up evaluation was executed with plain fluoroscopy or CAG (6%, 95% CI: 3–10%) (Figure 4).

### Type of included studies

**FIGURE 5:**
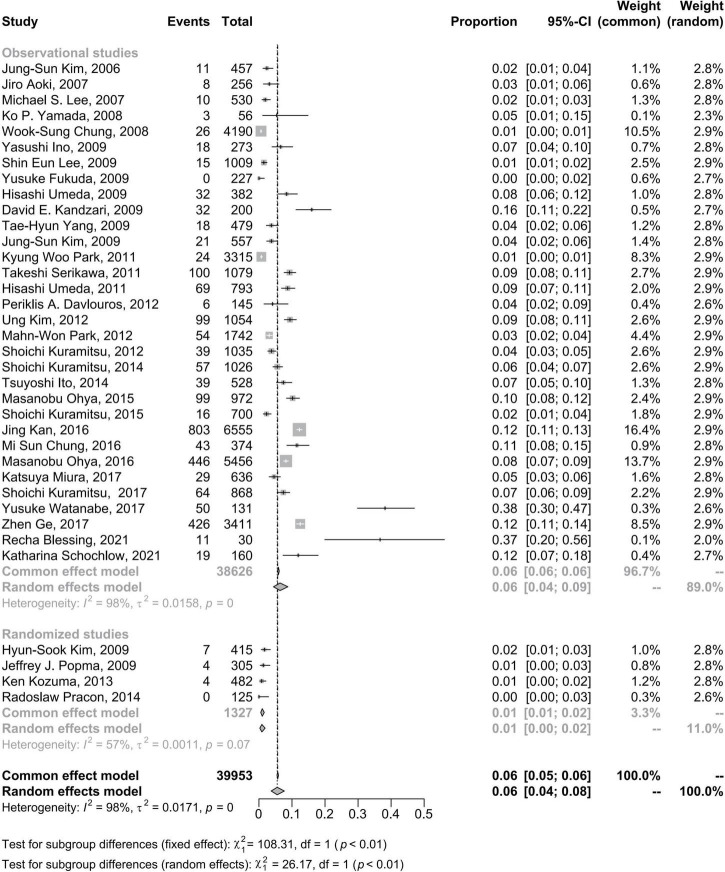
Forest plots of morbidity of coronary stent fracture in patient level in randomized and observational studies. CI, confidence interval.

Overall, the pooled estimate for the subgroup including randomized studies differed appreciably from the combined result from observational studies. The CSF pooled incidence in randomized studies was 1% (95% CI: 0–2%); in observational studies was 6% (95% CI: 4–9%) (Figure 5).

### Meta-regression

Generally, pulsation of the heart can result in metal fatigue and CSF. We evaluated the duration after stent implantation and the incidence of CSF with meta-regression. In the meta-regression analyses, patients’ overall CSF estimates were not modified by the duration of follow-up, which lasted for nearly 5 years (*P* = 0.8821, Table 4 and Supplementary Figure 4, Supplementary Table 4). The meta-regression results and scattered bubble plot of the incidence of CSF at the patient level between 2000 and 2020 showed a general upwards trend (*P* < 0.0001, Table 4 and Supplementary Figure 5, Supplementary Table 5). These data indicated that the CSF incidence rate increased from 2000 to 2020. Furthermore, the meta-regression analyses confirmed that RCA lesions were a risk factor for CSF incidence (Table 4). However, the analysis did not identify other potential effects for the incidence of CSF at the lesion level (Table 4).

**TABLE 4:** Results of meta-regression for morbidity of coronary stent fracture.

Covariate	Meta-regression coefficient	95% CI	*P*-value
Duration, month	–0.0005	–0.0067 to 0.0058	0.8821
Year collecting	0.0211	0.0130 to 0.0292	<0.0001
Age, year	0.0056	–0.0078 to 0.0191	0.4131
Male,%	0.4236	–0.1637 to 1.0109	0.1575
Hypertension,%	0.2103	–0.1415 to 0.5621	0.2414
Diabetes,%	–0.4722	–0.9547 to 0.0102	0.0550
Dyslipidemia,%	0.0181	–0.2096 to 0.2457	0.8763
RCA lesion,%	2.9486	0.5937 to 5.3035	0.0141
Type B2/C,%	0.0249	–0.0582 to 0.1079	0.5570
Stent length, mm	0.0021	–0.0071 to 0.0113	0.6532
Stent/lesion ratio	0.3181	–0.0302 to 0.6665	0.0735

RCA, right coronary artery.

### Sensitivity analyses

Fixed effects models did not change the results of CSF incidence at the patient level but had a slight impact on the CSF incidence at the lesion level and materially changed the results of CSF incidence at the stent level (Figure 2). In the sensitivity analysis using the R command metainf, there was no prominent change when omitting each included study separately at the patient level and lesion level but a relatively prominent change when omitting a study (7), at the stent level for CSF incidence (Supplementary Figures 6–8).

## Discussion

This systematic review and meta-analysis were conducted to estimate the incidence rates of CSF, characterize the epidemiology of CSF, compare CSF incidence rates between the different DES types and follow-up evaluation modes, and investigate the potential risk factors for the incidence of CSF. Several CSF characteristics were identified. First, the CSF incidence rate was not as low as expected (5.5% among patients, 4.8% among lesions, and 4.9% among stents). Second, the pooled CSF incidence rates of SES and EES were 6 and 2%, respectively. Third, the duration of the stent after stent implantation seemed to have no significant effect on the incidence of CSF. Fourthly, the incidence of CSF increased with time in recent decades. Finally, RCA lesions contributed significantly to CSF at the lesion level.

To our knowledge, coronary DES fracture was first reported in 2004 (51), and the first study (13) designed to explicitly investigate the incidence of coronary SES fracture occurred in 2007, with an estimate of 2.6% at the lesion level. Compared with the previous meta-analysis of CSF and combined with the meta-regression of our study, there was an increase in the incidence of CSF with time. On the one hand, the closed-cell design and stainless steel stent struts of first-generation DESs resulted in poor flexibility and conformability. However, second-generation stents, characterized by thinner struts, an open-cell design and increased radial strength, tolerated fatigue and reduced the incidence of CSF, as clinical studies have identified (6, 7, 52, 53). On the other hand, some new-generation DESs, such as Promus Element, had a significantly higher risk of longitudinal compression, especially when employed for ostial lesions (47). Furthermore, the incidence may have increased due to the expansion of revisit rates prompting the discovery of asymptomatic CSF and the extensive use of coronary stents in complex lesions (such as twisted angulation, calcification, and chronic total occlusion) in which there was need for longer stents, aggressive post-dilation, stents overlap or multiple stents implantation. Besides, advance in endovascular imaging technology and possibly regional specificity, demographic, sociocultural, and socioeconomic changes may also give rise to the increased incidence. Factors that may have contributed to a more revisit rates include progressive improvements in coverage of post-procedure education, or more widespread coverage of available medical resource.

Previous studies (15, 21, 39, 52, 54) have identified that CSF occurs more frequently in lesions treated with SES. There are some reasons for this. First, an inchoate SES is more likely to cause CSF because of its closed-cell design and stainless steel material with low flexibility and conformability (35). Second, compared with other stents, such as EESs, SESs are more prone to ISR (39, 55–57), which indicates a new hinge point in the stent. With cardiac impulse, the hinge point in the stent segment can be displaced or twisted, leading to stent fracture.

IVUS and OCT have been used as more sensitive methods for the detection of stent fracture due to their high resolution (16, 58, 59). In addition, multislice computed tomography (CT) (42) and CT angiography (27, 60, 61) have been identified as ideal imaging modalities for CSF than CAG. Because coronary stents are small in size and minimally radiopaque, and the 2D technique of CSF detection is viewing angle-dependent, the fractures occurring on one side of the stent could be missed (62). Additionally, an autopsy investigation showed a higher rate of DES fractures (29%) than that in normal clinical studies (53). This result implies that CSF may be underdiagnosed by fluoroscopy as a result of its limited sensitivity to detect stent fractures. However, there was no significant difference between fluoroscopy and IVUS/OCT/CT/CTA based on the current data. It needs to be emphasized that the results were affected by many issues, such as differences in diagnostic criteria, study population, duration of stent, stent types, management condition, and many hidden factors. Besides, not all patients in the group using OCT/IVUS were diagnosed with OCT/IVUS. Most studies stated that the IVUS/OCT was used when performer thought IVUS or OCT was in need. In fact, most follow-up evaluations were often performed using coronary angiography first, with IVUS or OCT being considered for complex lesions. Practically speaking, the number of patients in the group using CAG was much higher than the number of patients in the group using IVUS/OCT. The imbalanced sample size of the two groups may cause a potential bias and the small sample size of the group using IVUS/OCT may limit the power of the study to detect differences between the two groups. According to current knowledge, IVUS and OCT are still the gold standards for CSF diagnosis. Additionally, high heterogeneity was observed and this heterogeneity was still presented in subgroup analyses. More homogeneous studies need to be conducted in the future to explore the problem.

The quality control data of coronary stents has shown that stents can be used consistently for more than 10 years when they are expanded to the maximum diameter on the condition of simulative physiological heart beats (72 beats/min, 40 million beats/year), and no fatigue or fracture occurs. Combined with our analysis results of the association between the duration of the stent after stent implantation, it may be unnecessary for patients with stent implantation to undergo longer repeated evaluations for the occurrence of CSF. However, the best time range to detect CSF remains undefined.

In general, there is greater curvature and range of motion in the right coronary artery (RCA) than in the left coronary artery, especially in the proximal-to-middle segment of the RCA, indicating more severe cardiac motion and angulation, a classic risk factor for metal fatigue (63, 64). Additionally, overlapping stents, saphenous vein grafts, longer stent lengths, stainless stents and multiple stents are universally accepted to be related to CSF (13–15, 19, 54, 65–68). Among these, Kuramitsu et al. (35) revealed that hinge motion and tortuosity contributed the most to the incidence of CSF, suggesting that RCA lesions may be a more important risk factor for CSF.

However, there are some contradictions between our results and previous consensus. The indeterminate definition of CSF, stent type, study participants, incompleteness of follow-up and other factors in the included studies may account for the negative results of the meta-regression analysis of the association of follow-up modes, stent length and lesion type with the incidence of CSF.

Overall, our study findings support that the incidence of CSF has increased in recent years and that CSF remains an inevitable issue. Accordingly, it is vital for clinicians to be aware of the incidence and predictors of CSF. More importantly, it is useful to learn about the characteristics of stents for proper use of current DESs based on lesion characteristics, and an appropriate detection mode according to the complexity of the lesion is also essential. In addition, it seems unnecessary to carry out longer follow-ups to detect the occurrence of CSF.

This study has several limitations. First, studies including only participants with ST or ISR were excluded for their high heterogeneity, which may result in an underestimate of the incidence of CSF among all patients treated with stents, as CSF occurs more frequently among participants with ST or ISR. Second, there were few studies investigating the occurrence of CSF at the stent level, and the funnel plots showed an asymmetrical trend, which may indicate publication bias or be a result of small-study effects, that is, the tendency for smaller studies to show larger treatment effects. Third, even though we tried to account for the high heterogeneity with an inverse variance (DerSimonian–Laird) random effects model, it is difficult to interpret the results. Fourthly, analyses to assess the competing risk were not performed, meaning that the occurrence of the event could have been impacted by competing events. Finally, standards of diagnostic criteria for CSF were not established, and not all information was available, possibly leading to an underestimation of the incidence of CSF. Consequently, large prospective randomized trials are needed to better analyze and understand the phenomenon.

## Conclusion

CSF is a common phenomenon in patients treated with DES, and the incidence of CSF has increased in the past two decades. This meta-analysis provided reliable estimates of the incidence of CSF among patients with coronary heart disease treated with stent implantation. The point incidence of CSF was found to be 5.5% among patients, 4.8% among lesions, and 4.9% among stents, and the data had high heterogeneity. The results indicated that the duration of stent placement after stent implantation had no impact on the incidence of CSF. In addition, DES type and RCA lesions influenced the incidence of CSF. The potential mechanisms are multifactorial. It is important to understand the relevant features of stents and select appropriate stent types based on the type of lesion. Further research to design appropriate strategies and protocols for the monitoring, management, and prevention of CSF should be a matter of thorough investigation.

## Data availability statement

All datasets analyzed for this study are included in the article/Supplementary material.

## Author contributions

YC, DL, and RY conceived the study design and wrote the manuscript. YC, HL, XY, and JD performed the data collection and analyses. All authors read and approved the final manuscript.

## Footnotes

1
https://www.crd.york.ac.uk/prospero/display_record.php?ID=CRD42022311995
