# Supplementary material for: Incidence of coronary drug-eluting stent fracture: A systematic review and meta-analysis
Source: Front Cardiovasc Med. 2022 Aug 23;9:925912. doi: 10.3389/fcvm.2022.925912 (PMC9445981; doi:10.3389/fcvm.2022.925912)
Supplement: Supplementary file 1 [file Presentation_1.PDF]

Supplemental Data

Incidence of coronary drug-eluting stent fracture: a systematic review and meta-analysis

Yang Chen, MD; Dandan Li, MD; Yanhui Liao, MD; Xiongda Yao, MD; Yuehua Ruan, MD; Kai Zou, MD; Hanhui Liao, MD; Jingwen Ding, MD; Hao Qin, MD; Zuozhong Yu, MD; Yuanbin Zhao, MD; Longlong Hu, MD, PhD； Renqiang Yang, MD, PhD

Supplemental Figure

Figure S1. Risk of bias graph: review authors' judgements about each risk of bias item presented as percentages across all included studies pag.2
Figure S2. Risk of bias summary: review authors' judgements about each risk of bias item for each randomized included study..pag.2
Figure S3. Factors contributing to coronary stent fracture after stent implantation..................pag.3

Supplemental Tables

Table S1. PICOS criteria for inclusion and exclusion of studies into meta-analysis................pag.4
Table S2. Quality scoring for included papers using Newcastle-Ottawa Scale (NOS).............pag.5
Table S3. Synthesis of results..pag.6

Meta-regression

Figure S4. Scattered-plot of the relationship between stent duration and coronary stent fracture incidence...pag.7
Table S4. Meta-regression of the relation of stent duration to coronary stent fracture incidence...pag.7
Figure S5. Scattered-plot of the relationship between year collecting and coronary stent fracture incidence...pag.8
Table S5. Meta-regression of the relation of study year collecting to coronary stent fracture incidence...pag.8

Sensitivity Analysis

Figure S6. Sensitivity Analysis by calculating the combined coronary stent fracture incidence value and 95% confidence interval at the patient level after excluding each included study separately...pag.9
Figure S7. Sensitivity Analysis by calculating the combined coronary stent fracture incidence value and 95% confidence interval at the lesion level after excluding each included study separately...pag.10
Figure S8. Sensitivity Analysis by calculating the combined coronary stent fracture incidence value and 95% confidence interval at the stent level after excluding each included study separately...pag.10

Assessment of publication bias for Randomized Controlled Trials.................................pag.11

Publication bias

Figure S9. Funnel plot of coronary stent fracture incidence in patient level..........................pag.14
Figure S10. Egger’s funnel plot of coronary stent fracture incidence in patient level............pag.14
Table S6. Egger’s test for coronary stent fracture incidence in patient level..........................pag.14
Figure S11. Funnel plot of coronary stent fracture incidence in lesion level..........................pag.15
Figure S12. Egger’s funnel plot of coronary stent fracture incidence in lesion level.............pag.15
Table S7. Egger’s test for coronary stent fracture incidence in lesion level...........................pag.15

Figure S13. Funnel plot of coronary stent fracture incidence in stent level pag.16
Figure S14. Egger's funnel plot of coronary stent fracture incidence in stent level...............pag.16
Table S8. Egger's test for coronary stent fracture incidence in stent level.............................pag.16

Supplemental Figure

Figure S1. Risk of bias graph: review authors' judgements about each risk of bias item presented as percentages across all included studies.

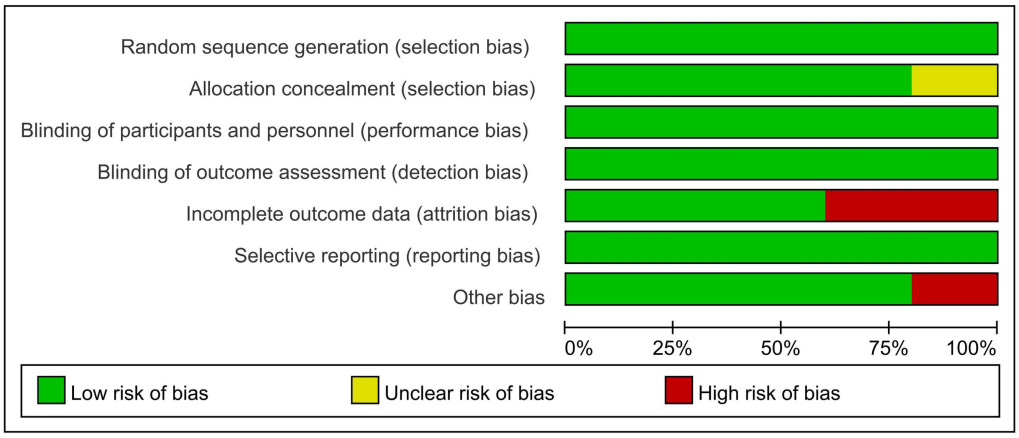

Figure S2. Risk of bias summary: review authors' judgements about each risk of bias item for each randomized included study.

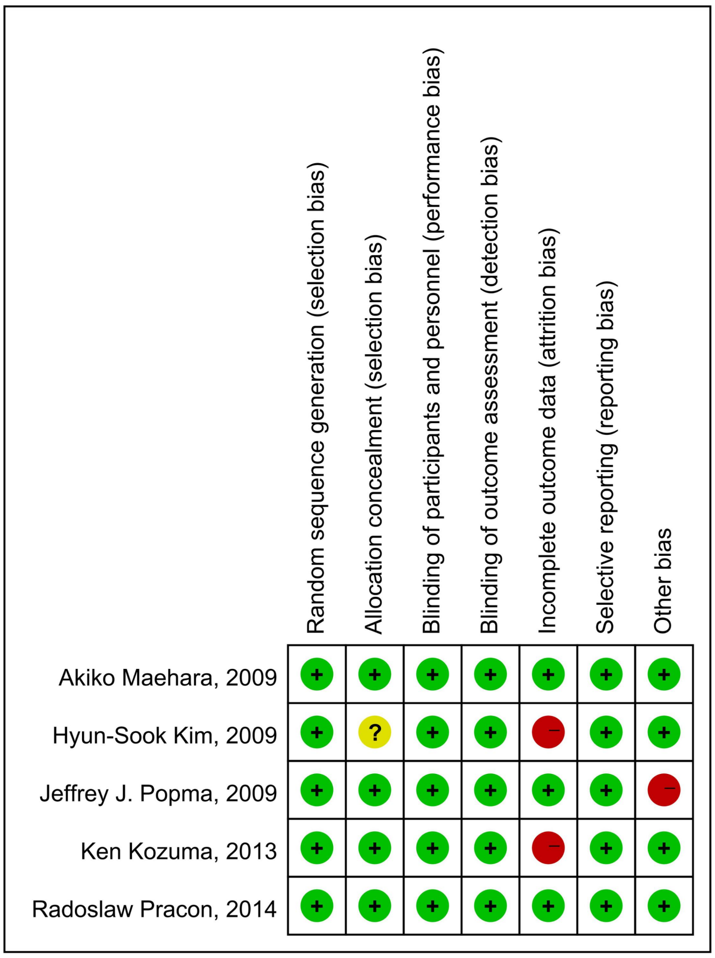

Figure S3. Factors contributing to coronary stent fracture after coronary drug-eluting stent implantation.

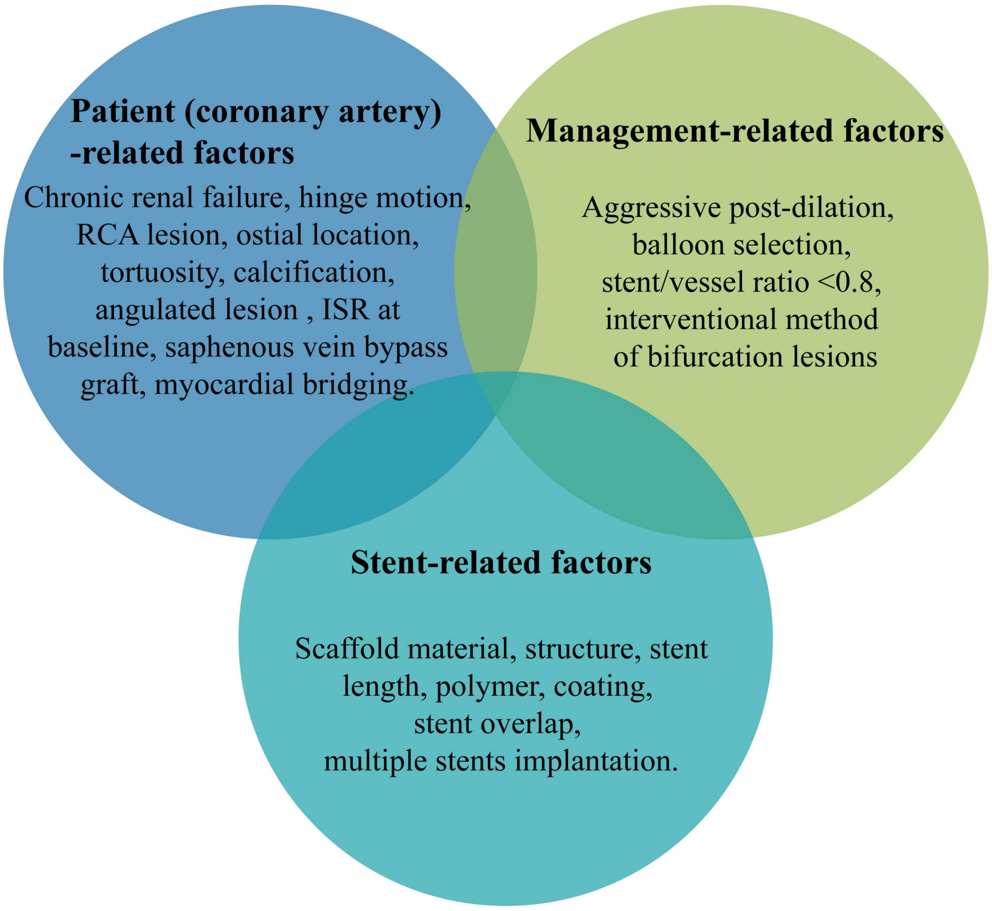

Supplemental tables

Table S1. PICOS Criteria for Inclusion and Exclusion of Studies into Qualitative/Quantitative Meta-analysis

Parameter	Inclusion criteria	Exclusion criteria
Patients	Patients with coronary heart disease who received successful drug eluting stent (DES) implanation and follow-up fluoroscopy/coronary angiography (CAG)/plain fluoroscopy/intravascular ultrasound (IVUS)/optical coherence tomography(OCT) evaluation.	Age ≤18 years; with a planned surgery requiring antiplatelet therapy withdrawal; intolerance of aspirin or ticlopidine/clopidogrel; inappropriate vessel size for DES; vitro research; animal models; autopsy; included population were patients with in-stent restenosis (ISR) or stent thrombosis (ST)
Intervention	Patients receiving successful DES implanation	-
Comparator	Patients with coronary stent fracture (CSF)<br>Patients without CSF	-
Outcomes	CSF	-
Study design	Clinical randomized trials<br>Controlled before-and-after studies<br>Case control study<br>Prospective and retrospective cohort studies	Cross-sectional studies<br>Repeat publications of the same analysis or dataset<br>Case reports<br>Conference abstracts<br>Opinion pieces<br>Books or grey literature

Abbreviations: CAG, coronary angiography; CSF, coronary stent fracture; DES, drug eluting stent; ISR, in-stent restenosis; IVUS, intravascular ultrasound; MI, myocardial infarction; OCT, optical coherence tomography; PICOS, patients, intervention, comparator, outcomes, study design; ST, stent thrombosis.

Table S2. Quality scoring for included cohort studies using Newcastle-Ottawa Scale

Study (Author, Year)	Selection	Comparability	Outcome	Exposure	*Total*
Jung-Sun Kim, 2006	4	2	1	–	*7*
Masanori Okumura, 2007	4	2	3	–	*8*
Jiro Aoki, 2007	4	2	3	–	*9*
Michael S. Lee, 2007	4	1	2	–	*7*
Ko P. Yamada, 2008	4	0	3	–	*7*
Wook-Sung Chung, 2008	4	1	2	–	*7*
Yasushi Ino, 2009	4	2	3	–	*9*
Harvey S. Hecht, 2009	3	1	2	–	*6*
Shin Eun Lee, 2009	4	2	1	–	*7*
Yusuke Fukuda, 2009	3	1	3	–	*7*
Hisashi Umeda, 2009	4	2	3	–	*9*
David E. Kandzari, 2009	3	2	3	–	*8*
Tae-Hyun Yang, 2009	4	2	2	–	*8*
Jung-Sun Kim, 2009	4	2	2	–	*8*
Yasushi Ino, 2010	3	2	3	–	*8*
Tomoko Kawai, 2010	4	1	3	–	*8*
Sang Min Park, 2011	4	2	2	–	*8*
Kyung Woo Park, 2011	4	2	2	–	*8*
Takeshi Serikawa, 2011	4	2	2	–	*8*
Hisashi Umeda, 2011	4	2	2	–	8
Yasushi Ino, 2011	3	2	3	–	*8*
Periklis A. Davlouros, 2012	4	2	2	–	*8*
Ung Kim, 2012	4	2	2	–	*8*
Mahn-Won Park, 2012	4	2	2	–	*8*
Shoichi Kuramitsu, 2012	4	2	3	–	*9*
Diaa A. Hakim, 2013	2	2	2	–	*6*
Hironori Hara, 2013	3	2	3	–	*8*
Shinji Inaba, 2014	4	0	2	–	*6*
Shoichi Kuramitsu, 2014	4	2	2	–	*8*
Tsuyoshi Ito, 2014	4	2	2	–	*8*
Masanobu Ohya, 2015	4	2	3	–	*9*
Shoichi Kuramitsu, 2015	4	2	3	–	*9*
Jing Kan, 2016	4	2	2	–	*8*
Mi Sun Chung, 2016	3	2	2	–	*7*
Masanobu Ohya, 2016	4	2	3	–	*9*
Katsuya Miura, 2017	3	2	2	–	*7*
Shoichi Kuramitsu, 2017	4	2	2	–	*8*
Yusuke Watanabe, 2017	4	2	3	–	*9*
Zhen Ge, 2017	4	2	2	–	*8*
Recha Blessing, 2021	3	2	3	–	*8*
Katharina Schochlow, 2021	3	2	2	–	*7*

A study can be awarded a maximum of 4 points for the Selection category, 2 points for the comparability category and 3 points for the Outcome/Exposure categories. Therefore, the maximum points a study can obtain is 9 which indicates a high-quality study.

Table S3. Synthesis of results

Outcomes	Analysis	Included studies	Pooled Incidence	95% CI	Events/Population	Hetereogenity (%)	*p*
**Incidence of coronary stent fracture in patient level**	Inverse variance (DerSimonian – Laird) random-effects mode	36	0.055	0.037–0.077	2702 /39953	98	**< 0.001**
**Incidence of coronary stent fracture in lesion level**	Inverse variance (DerSimonian – Laird) random-effects mode	29	0.048	0.031 – 0.068	3188/39945	99	**< 0.001**
**Incidence of coronary stent fracture in stent level**	Inverse variance (DerSimonian – Laird) random-effects mode	8	0.049	0.025–0.094	3757/19252	98	**< 0.001**

Meta-regression

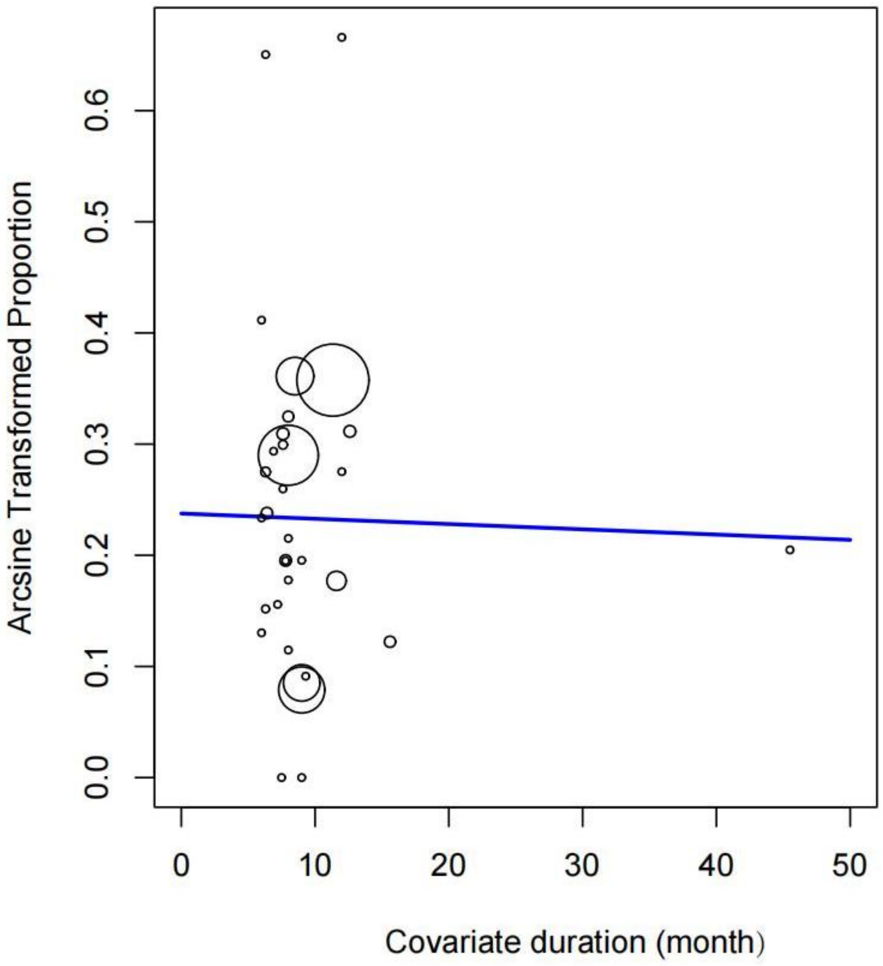

Figure S4. Scattered-plot of the relationship between stent duration and coronary stent fracture incidence

Table S4. Meta-regression of the relation of stent duration to coronary stent fracture incidence

Covariate	Estimate	Lower bound	Upper bound	Std. error	p-Value
**Intercept.**	0.2376	0.1649	0.3157	0.0371	<0.0001
**Duration**	-0.0005	-0.0067	0.0058	0.0032	0.8821

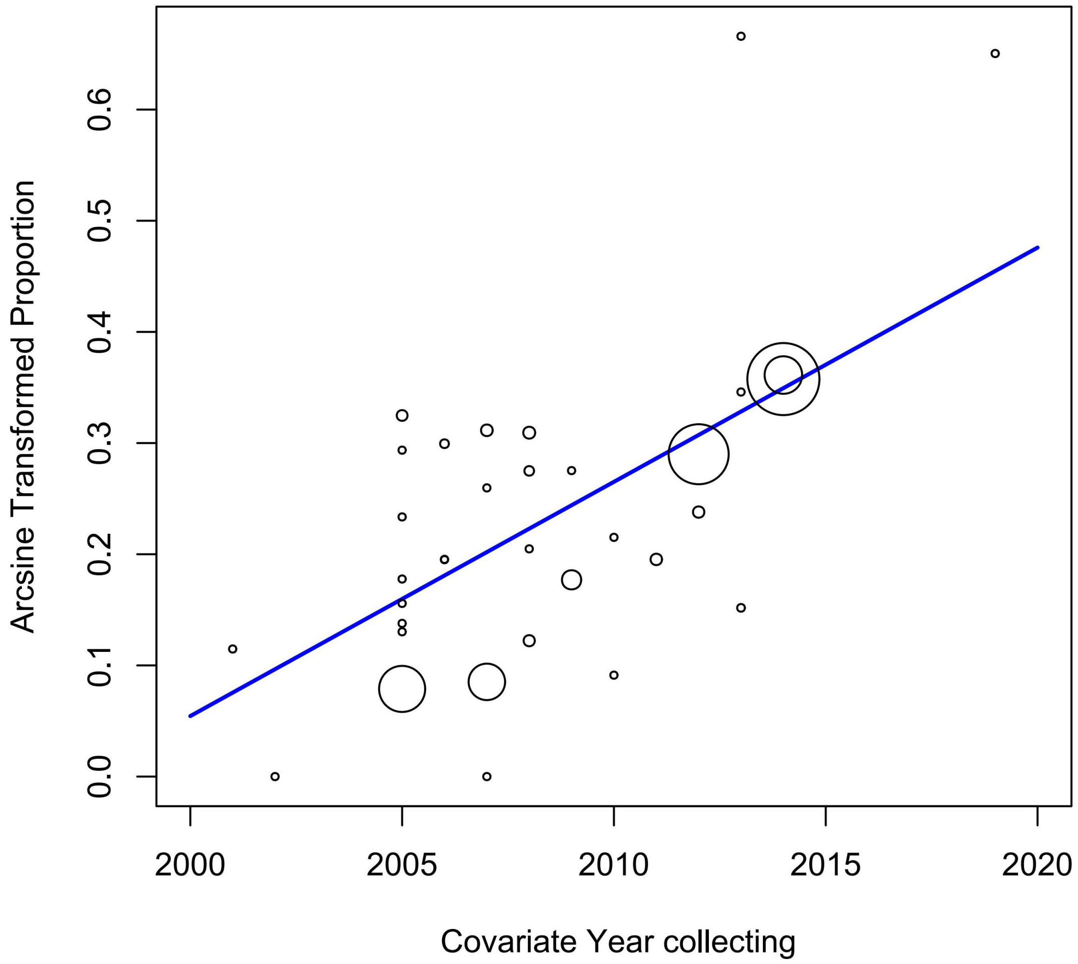

Figure S5. Scattered-plot of the relationship between year collecting and coronary stent fracture incidence

Table S5. Meta-regression of the relation of study year collecting to coronary stent fracture incidence

Covariate	Estimate	Lower bound	Upper bound	Std. error	p-Value
**Intercept.**	-42.0857	-58.3365	-25.8349	8.2914	<0.0001
**Year collecting**	0.0211	0.0130	0.0292	0.0041	<0.0001

Sensitivity Analysis

Figure S6. Sensitivity Analysis by calculating the combined coronary stent fracture incidence value and 95% confidence interval at the patient level after excluding each included study separately

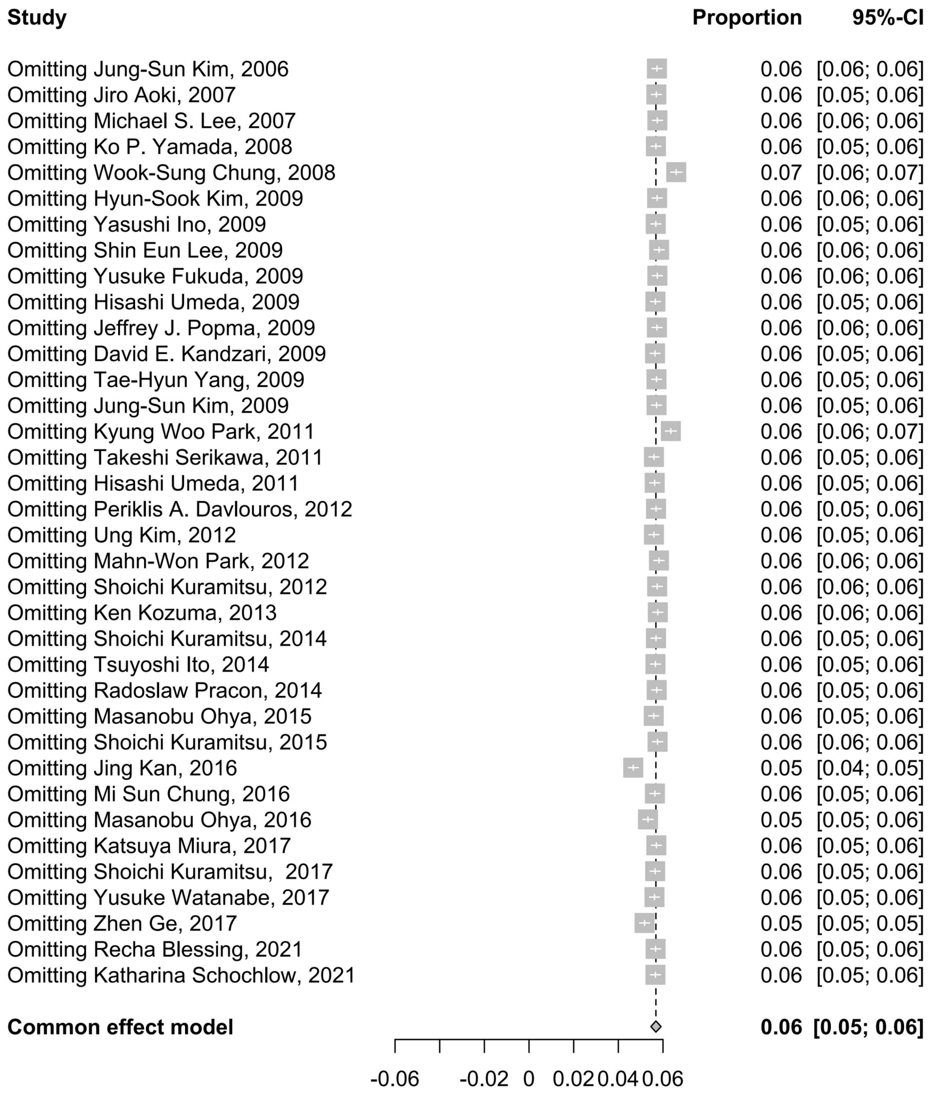

Figure S7. Sensitivity Analysis by calculating the combined coronary stent fracture incidence value and 95% confidence interval at the lesion level after excluding each included study separately

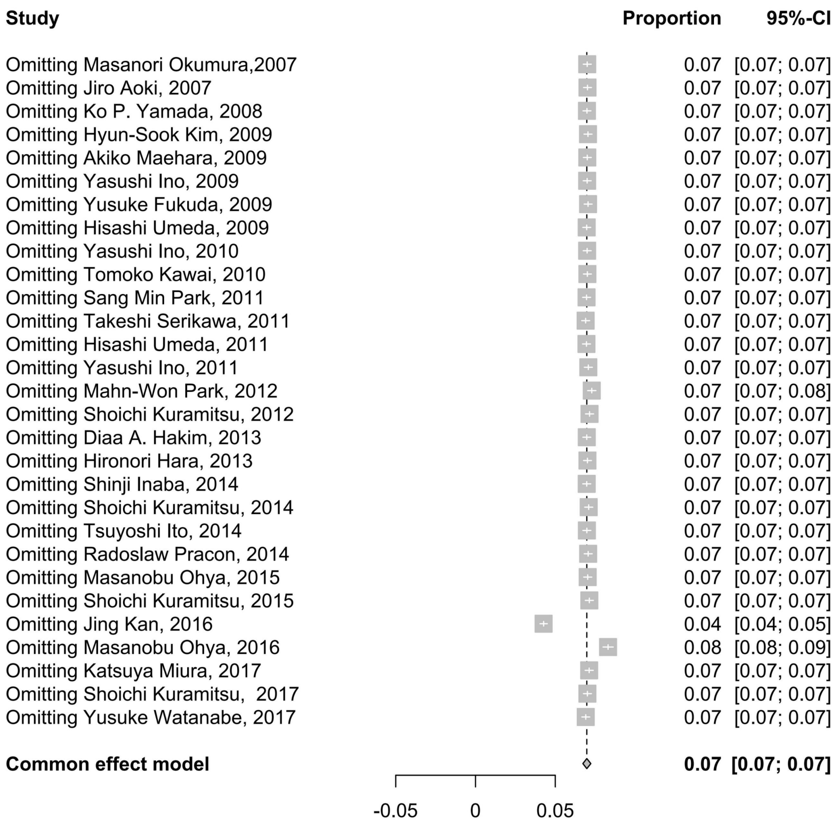

Figure S8. Sensitivity Analysis by calculating the combined coronary stent fracture incidence value and 95% confidence interval at the stent level after excluding each included study separately

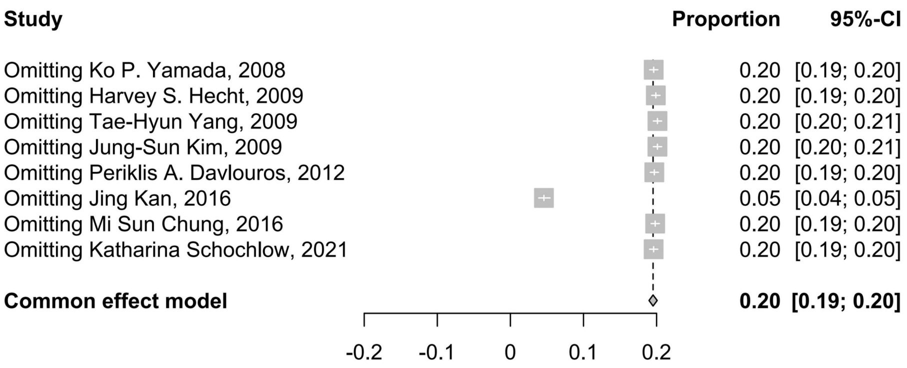

Assessment of publication bias for RCT

Hyun-Sook Kim, 2009

Domain	Support for judgement	Risk of bias
**Random sequence generation**	Computer-generated randomization sequence	Low
**Allocation concealment**	No sufficient details	Unclear
**Participant/personnel blinding**	No blinding, but the review authors judge that the outcome is not likely to be influenced by lack of blinding	Low
**Outcome assessor blinding**	All adverse clinical events were adjudicated by an independent events committee blinded to the treatment groups	Low
**Incomplete outcome data**	Missing partial repeat angiography	High
**Selective reporting**	All outcome specified in the methods reported in the results. Appears free of selection bias	Low
**Other bias**	Independent funding	Low

Jeffrey J. Popma, 2009

Domain	Support for judgement	Risk of bias
**Random sequence generation**	Automated telephone randomization system	Low
**Allocation concealment**	Stents were identical in appearance	Low
**Participant/personnel blinding**	Double blind	Low
**Outcome assessor blinding**	Clinical events were adjudicated by an independent events committee blinded to the treatment groups	Low
**Incomplete outcome data**	Partial patients withdrawn from the study, but the review authors judge that the reason of the withdrawn is not likely to be influenced.	Low
**Selective reporting**	All outcome specified in the methods reported in the results. Appears free of selection bias	Low
**Other bias**	Supported by related company	High

Akiko Maehara, 2009

Domain	Support for judgement	Risk of bias
**Random sequence generation**	Telephone randomization was performed with the use of a computerized, interactive voice response system.	Low
**Allocation concealment**	Open-label, but the review authors judge that the outcome is not likely to be influenced the allocation.	Low
**Participant/personnel blinding**	No blinding, but the review authors judge that the outcome is not likely to be influenced by lack of blinding	Low
**Outcome assessor blinding**	No blinding, but the review authors judge that the outcome is not likely to be influenced by lack of blinding	Low
**Incomplete outcome data**	Partial patients withdrawn from the study, but the review authors judge that the reason of the withdrawn is not likely to be influenced.	Low
**Selective reporting**	All outcome specified in the methods reported in the results. Appears free of selection bias	Low
**Other bias**	Independent funding	Low

Ken Kozuma, 2013

Domain	Support for judgement	Risk of bias
**Random sequence generation**	No details	Low
**Allocation concealment**	Assignments were blinded to the statistician, members of the independent clinical events committee, steering committee, academic research organization, and the sponsor	Low
**Participant/personnel blinding**	Single blind	Low
**Outcome assessor blinding**	No blinding of outcome assessment, but the review authors judge that the outcome measurement is not likely to be influenced by lack of blinding	Low
**Incomplete outcome data**	Missing partial repeat angiography	High
**Selective reporting**	All outcome specified in the methods reported in the results. Appears free of selection bias	Low
**Other bias**	Independent funding	Low

Radoslaw Pracon, 2014

Domain	Support for judgement	Risk of bias
**Random sequence generation**	Pseudorandom number generator	Low
**Allocation concealment**	Results were reviewed by independent institution without knowledge of the patient allocation.	Low
**Participant/personnel blinding**	Double blind	Low
**Outcome assessor blinding**	Events were reviewed and adjudicated independent by an committee whose members were unaware of the patients' treatment allocation.	Low
**Incomplete outcome data**	Partial patients were lost to follow up. The ratio of lost to follow up were reported and the review authors judge that the outcome was not likely to be influenced.	Low
**Selective reporting**	All outcome specified in the methods reported in the results. Appears free of selection bias	Low
**Other bias**	Independent funding	Low

Publication bias

Figure S9. Funnel plot of coronary stent fracture in patient level

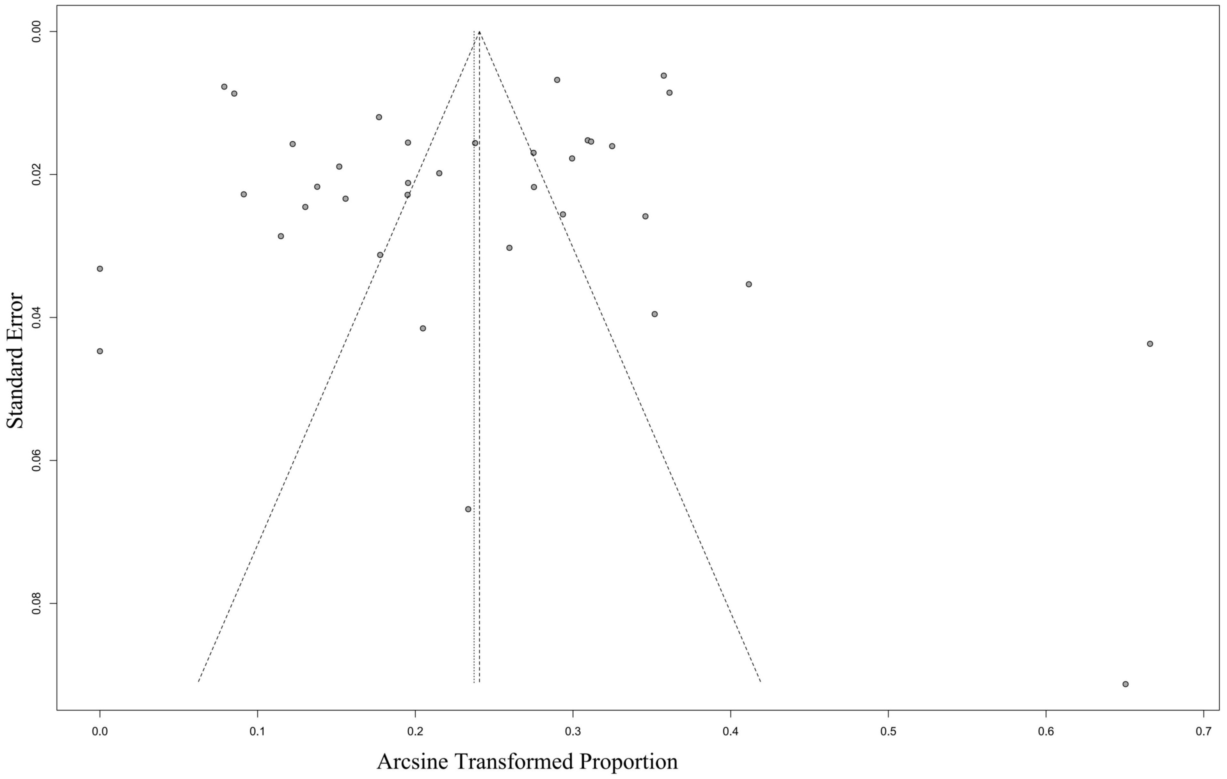

Figure S10. Egger’s funnel plot of coronary stent fracture incidence in patient level

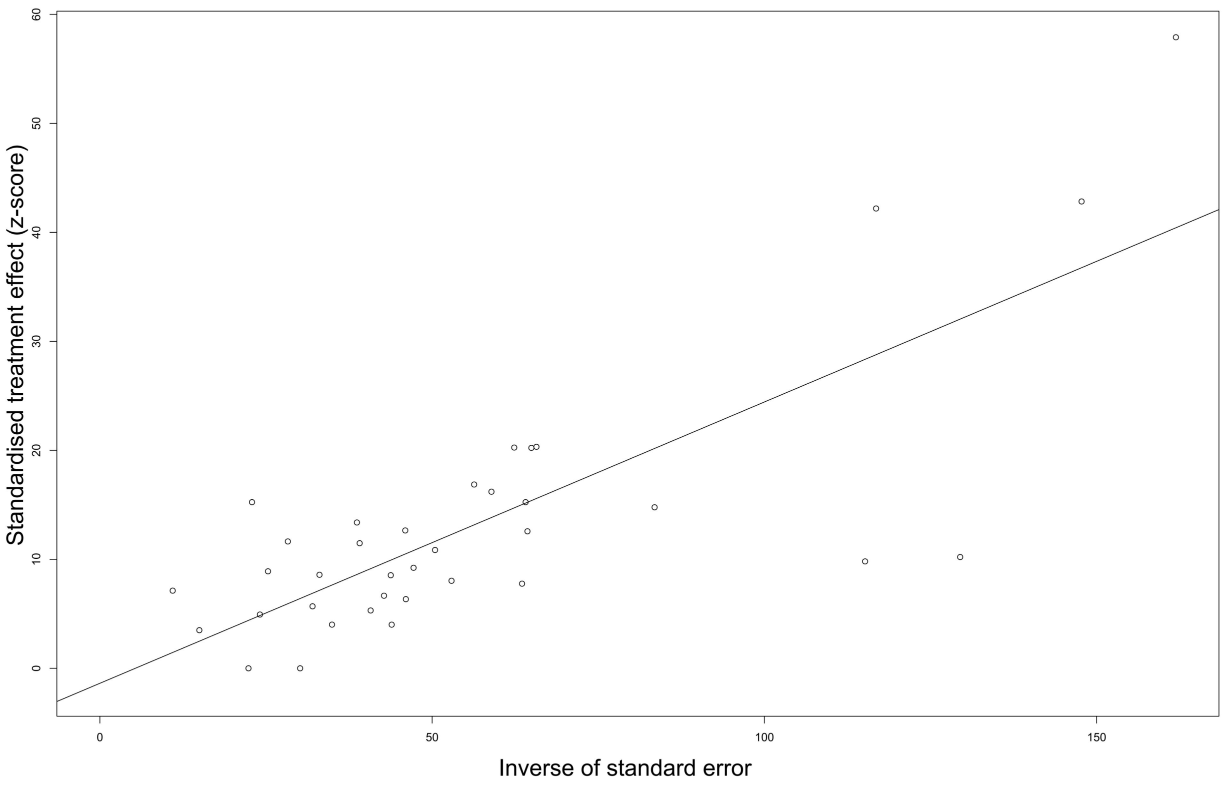

Table S6. Egger’s test for coronary stent fracture incidence in patient level

t	df	P-value
-0.59	34	0.5619

Figure S11. Funnel plot of coronary stent fracture incidence in lesion level

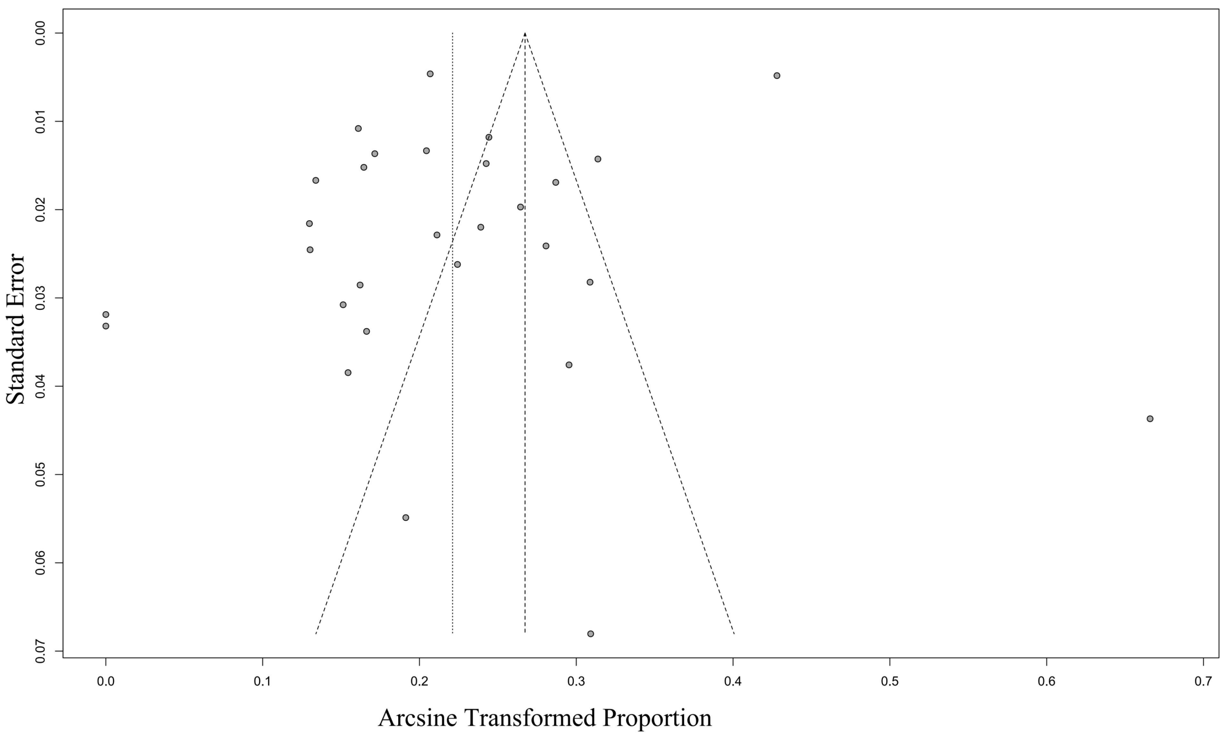

Figure S12. Egger's funnel plot of coronary stent fracture incidence in lesion level

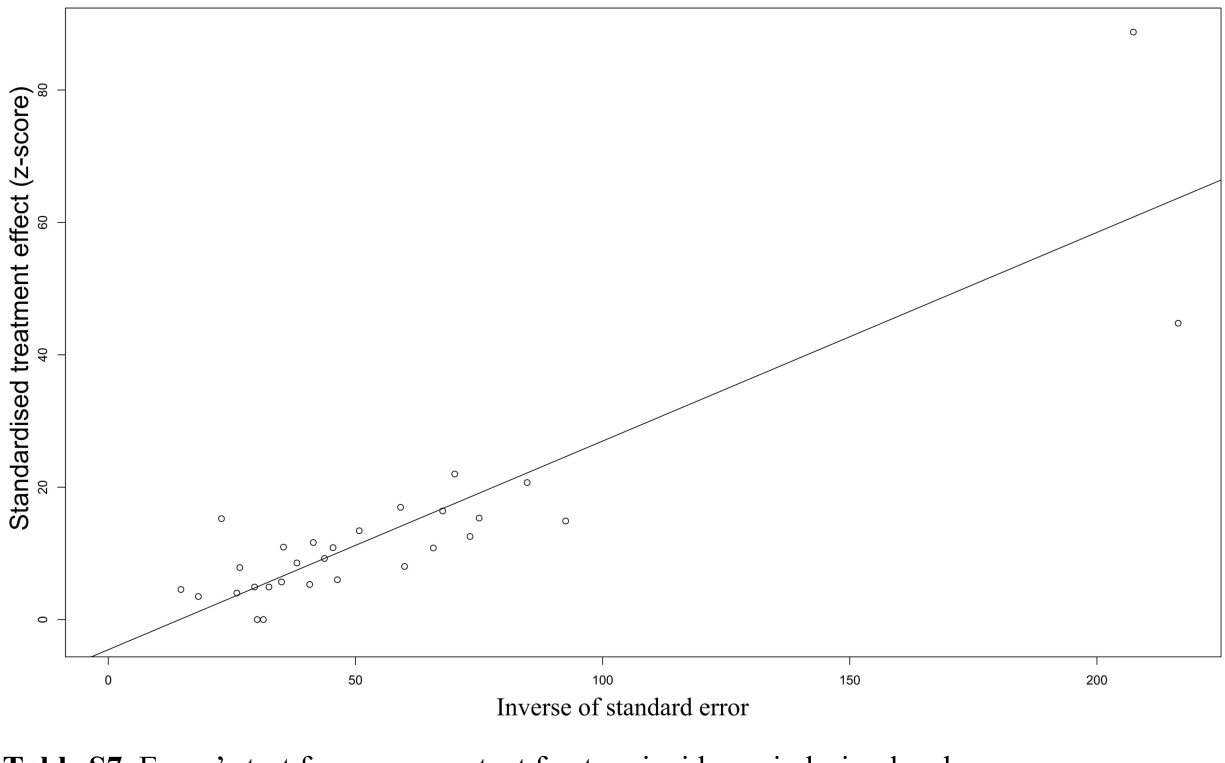

Table S7. Egger's test for coronary stent fracture incidence in lesion level

t	df	P-value
-1.93	27	0.0639

Figure S13. Funnel plot of coronary stent fracture incidence in stent level

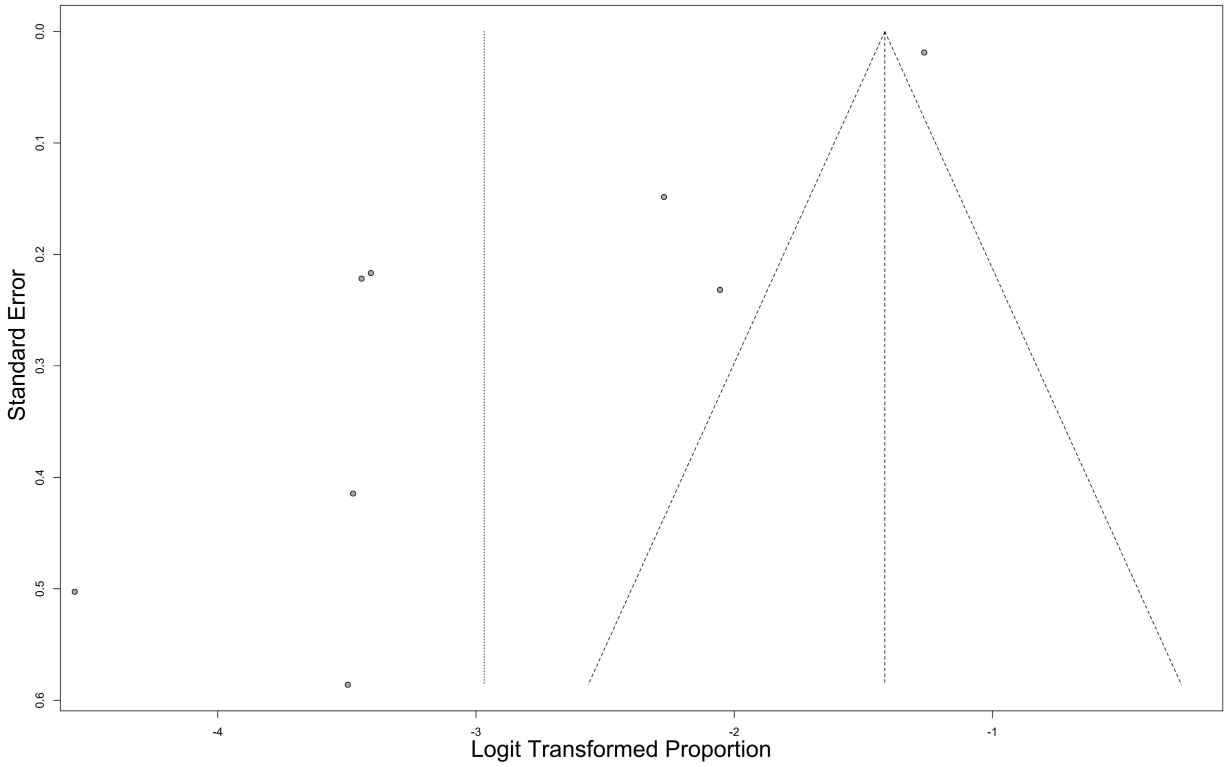

Figure S14. Egger's funnel plot of coronary stent fracture incidence in stent level

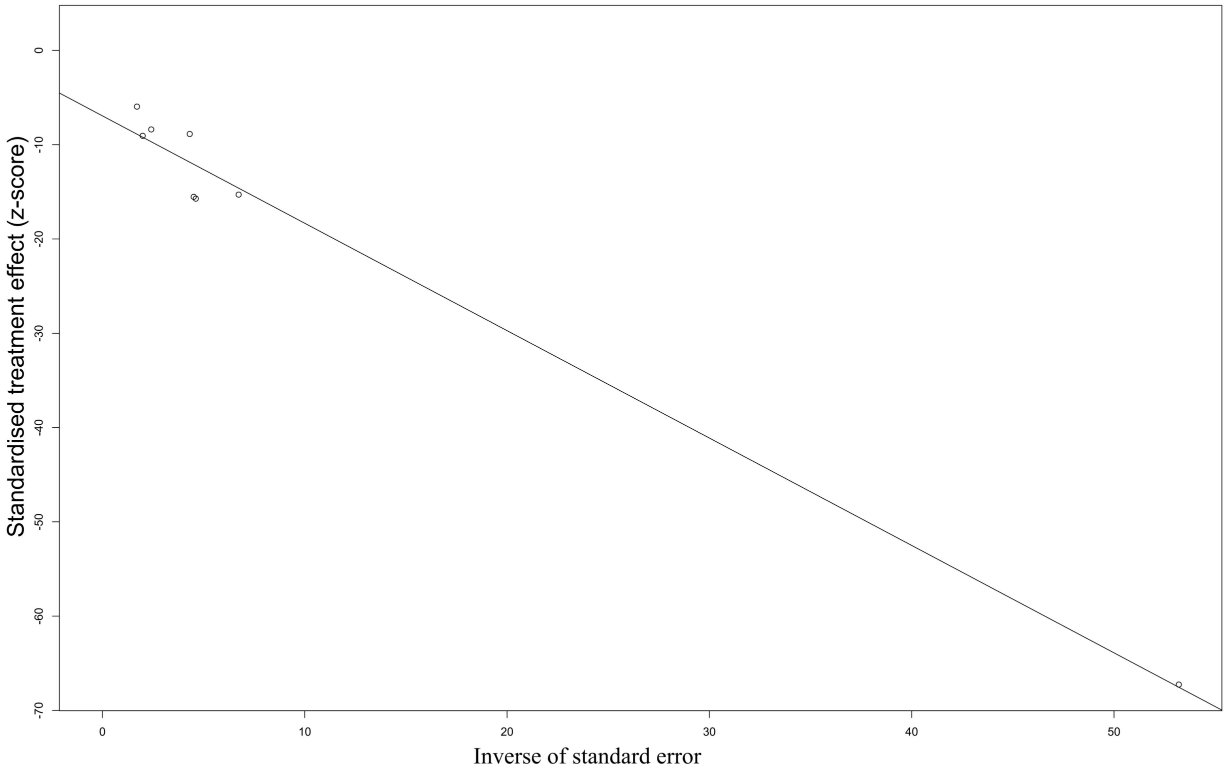

Table S8. Egger's test for coronary stent fracture incidence in stent level

t	df	P-value
-6.20	6	0.0008
